# Supplementary material for: Mechanistic studies of a lipase unveil effect of pH on hydrolysis products of small PET modules
Source: Nat Commun. 2023 Jun 15;14:3556. doi: 10.1038/s41467-023-39201-1 (PMC10272158; doi:10.1038/s41467-023-39201-1)
Supplement: Supplementary file 1 — Supplementary Information [file 41467_2023_39201_MOESM1_ESM.pdf]

Supplementary Information

Mechanistic studies of a lipase unveil effect of pH on hydrolysis products of small PET modules

Katarzyna Świderek,[1,*] Susana Velasco-Lozano,[2] Miquel À. Galmés,[1] Ion Olazabal,[3] Haritz Sardon,[3] Fernando López-Gallego,[2,4,*] Vicent Moliner[1,*]

1. BioComp Group, Institute of Advanced Materials (INAM), Universitat Jaume I, 12071 Castellón, Spain.
2. Heterogeneous Biocatalysis Laboratory, Center for Cooperative Research in Biomaterials (CIC biomaGUNE), Basque Research and Technology Alliance (BRTA), Donostia San Sebastián, Spain.
3. POLYMAT, Department of Polymer Science and Technology, University of the Basque Country UPV/EHU, Manuel de Lardizabal, 3, 20018, Donostia, Spain.
4. IKERBASQUE, Basque Foundation for Science, 48013 Bilbao, Spain.

Content

Supplementary Fig.1. SDS-PAGE gel 4

Supplementary Fig.2. ^{1}H NMR BHET 5

Supplementary Fig.3. Time courses of BHET hydrolysis with pH-controlled product formation 6

Supplementary Fig.4. UPLC-MS analysis of sample reactions at different pHs using pure BHET. 7

Supplementary Fig.5. Kinetic parameters of soluble CALB 8

Supplementary Fig.6. ^{1}H NMR enzymatic reaction sample at pH 5 using crude BHET 9

Supplementary Fig.7. ^{1}H NMR enzymatic reaction sample at pH 9 using crude BHET 9

Supplementary Fig.8. UPLC-MS analysis of sample reaction at pH 5 or 9 from crude BHET .. 10

Supplementary Fig.9. Analysis of hydrolysis of a PET trimer at pH 5 or 9. 11

Supplementary methods 12

Supplementary Fig.10. Schematic representation of the QM sub-set region 14

Supplementary Table 1. Atom types, charges (in a.u.) and parameters obtained after parametrization of BHET 18

Supplementary Table 2. Atom types, charges (in a.u.) and parameters obtained after parametrization of MHET 19

Supplementary Table 3. Atom types, charges (in a.u.) and parameters obtained after parametrization of MHET$^{(-)}$ 19

Supplementary Fig.11. pKa values of the titratable residues of CALB as derived from the PropKa ver. 3.0 3. 21

Supplementary Fig.12. Titratable curves for Lys residues (top panel), Glu residues (center panel) and Asp residues (bottom panel) of CALB 22

Supplementary Fig.13. Geometrical analysis of the pattern of interactions established between Glu294 and its surroundings at pH 5 and pH 9. 23

Supplementary Fig.14. Geometrical analysis of the pattern of interactions established between Lys136 and its surroundings at pH 5 and pH 9. 23

Supplementary Fig.15. Geometrical analysis of the pattern of interactions established between Glu81 and its surroundings at pH 5 and pH 9. 24

Fig 16. Geometrical analysis of the pattern of interactions established between Gln157 and its surroundings at pH 5 and pH 9. 24

Supplementary Fig.17. Interactions between BHET and CALB 25

Supplementary Fig.18. Interactions between MHET$^{(-)}$ and CALB 25

Supplementary Fig.19. Stability of MHET:CALB complex systems in WT and D134A variant. 26

Supplementary Fig.20-22. M06-2X:AM1/MM free energy surfaces 27

Supplementary Fig.23. Schematic representation of the reaction mechanism of the hydrolysis of the BHET/MHET catalysed by CALB........ 30
Supplementary Table 4. M06-2X:AM1/MM relative free energies........ 31
Supplementary Table 5. Key inter-atomic distances (in Å) of structures optimized at M06-2X:AM1/MM level........ 32
Supplementary Table 6: ESP atomic charges........ 33
Supplementary Table 7. Cartesian coordinates of TSs of the hydrolysis of BHET at pH 5 34
Supplementary Table 8. Cartesian coordinates of TSs of the hydrolysis of BHET at pH 9 36
Supplementary Table 9. Cartesian coordinates of TSs of the hydrolysis of MHET(-) at pH 5 . 38
Supplementary References........ 40

Experimental results

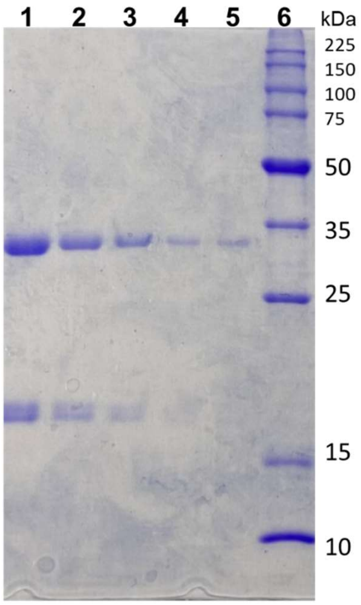

Supplementary Fig.1. SDS-PAGE gel containing different concentrations of commercial preparation of CALB (33 kDa) and the molecular weight marker with known protein concentration (Promega). Lanes: 1 – 5 CALB (Dilutions 10, 20, 40, 80_a and 80_b, respectively), lane 6: molecular weight marker, all protein bands correspond to 0.1 mg/mL of protein unless 50 kDa band which contains 0.3 mg/mL. This gel is representative of one gel independently prepared.

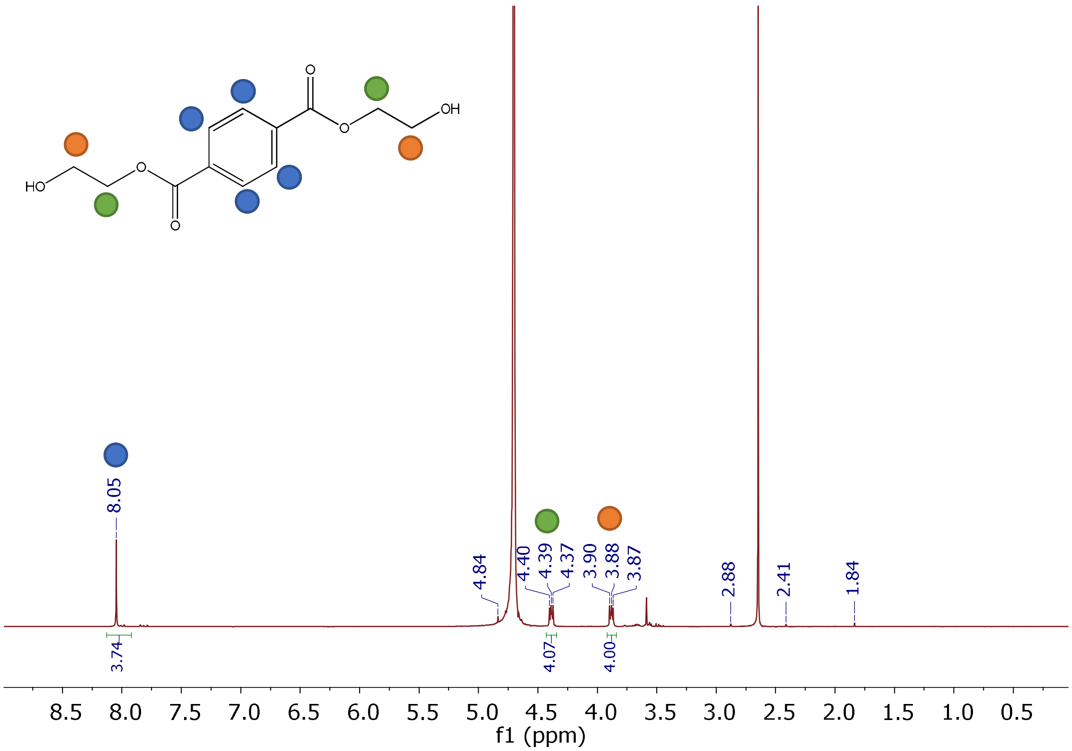

Supplementary Fig.2. ^{1}H NMR BHET on water: (300 MHz, Deuterium Oxide) δ 8.05 (s, 4H), 4.39 (t, 4H), 3.88 (t, 4H). Commercial BHET purchased from Sigma Aldrich and purified BHET from the organocatalytic depolymerization were indistinguishable. Source data are provided as a Source Data file.

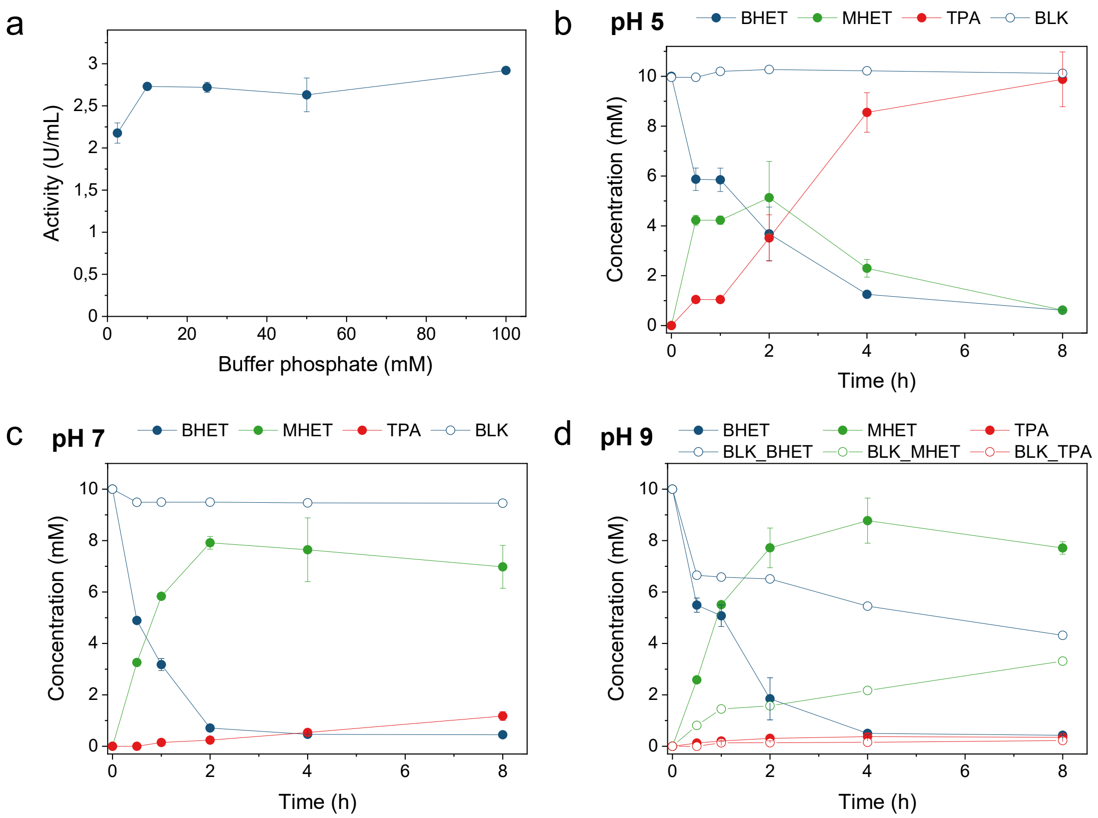

Supplementary Fig.3. a. CALB activity at different ionic strengths. The activity assay was performed with 0.5 mM of p-nitrophenyl butyrate in 10-100 mM sodium phosphate at pH 7 using the same enzyme concentration. **b-d.** Time courses of BHET hydrolysis with pH-controlled product formation.: **b,** pH 5, **c,** pH 7, and **d,** pH 9. Filled circles represent enzymatic reaction products, while empty circles represent reaction products in the absence of the enzyme (blanks). In all cases, CALB (5.12 mg/mL) was mixed with 10 mM BHET in 100 mM buffered aqueous solution containing 10% DMSO and the reaction mix was incubated at 25 ºC. Data are the mean value of n=2 independent measurements and error bars mean the standard deviation of the independent duplicates. Source data are provided as a Source Data file.

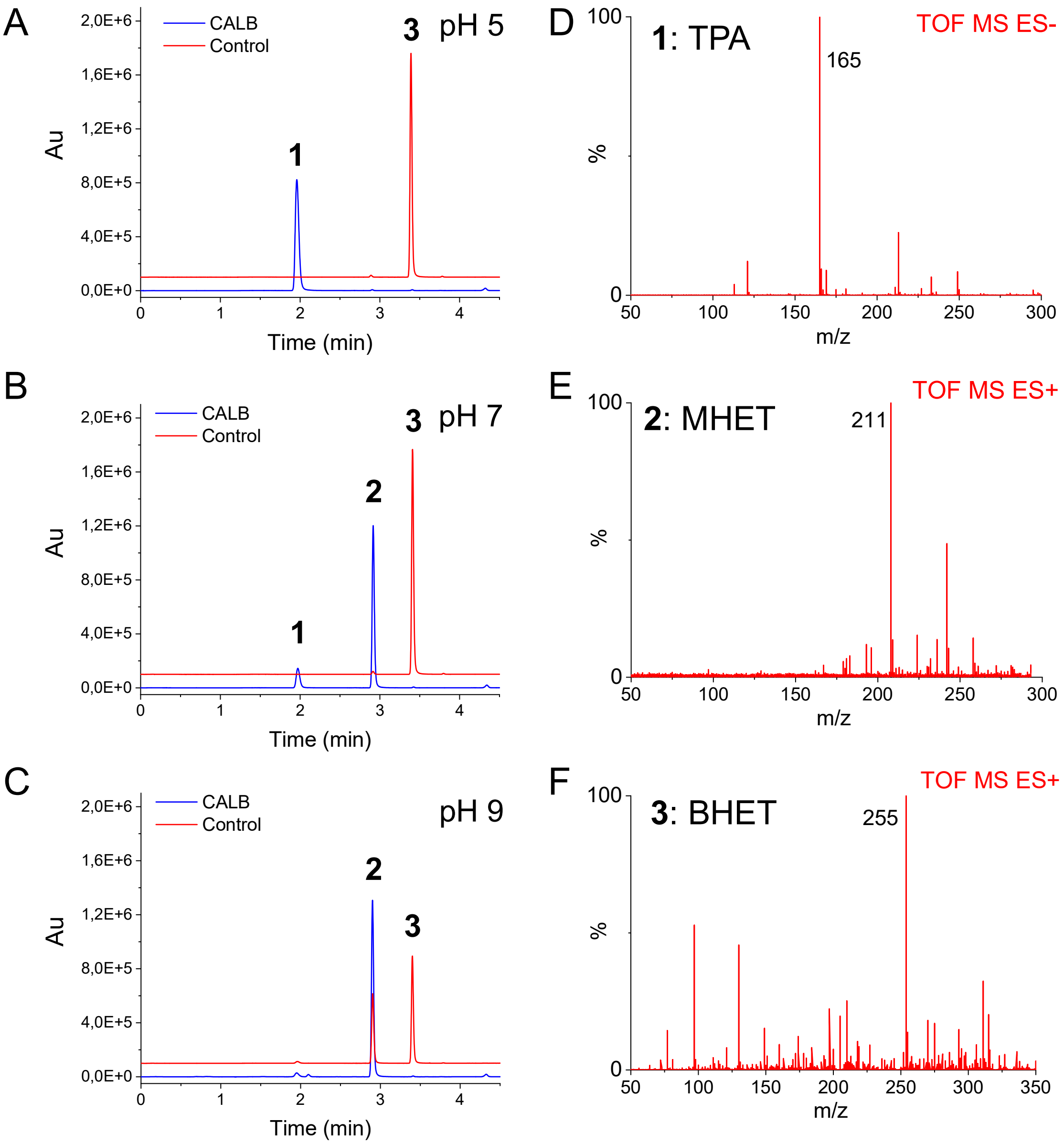

Supplementary Fig.4. UPLC-MS analysis of sample reactions at different pHs using pure BHET. Reaction with enzyme (blue line), reaction control without enzyme (red line). A) pH 5, B) pH 7 and C) pH 9. UPLC-MS spectra: D) MS analysis of peak 1, retained at 1.97 min corresponding to TPA. E) MS analysis of peak 2, retained at 2.92 min corresponding to MHET. F) MS analysis of peak 3, retained at 3.41 min corresponding to BHET. Source data are provided as a Source Data file.

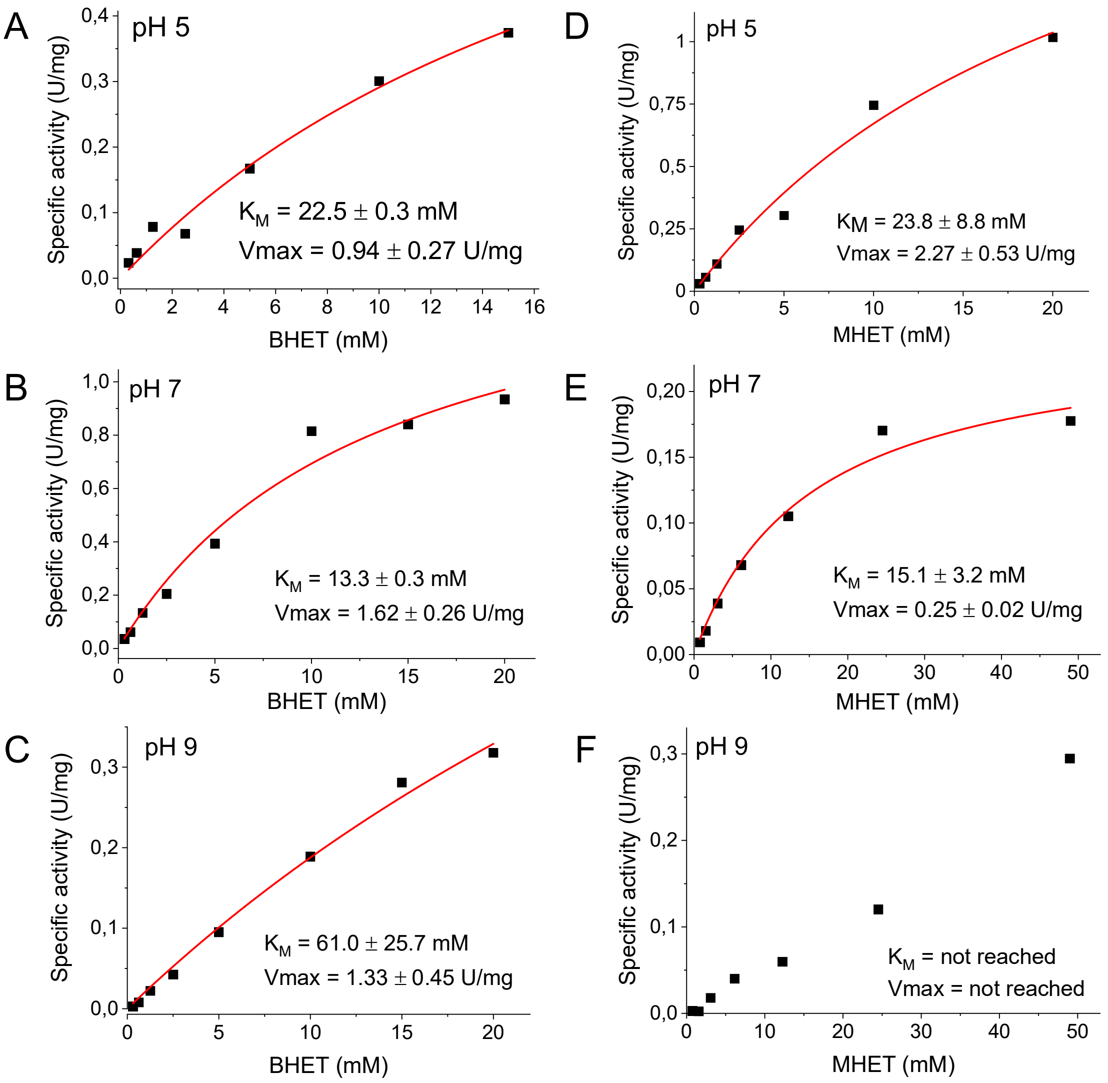

Supplementary Fig.5. Kinetic parameters of soluble CALB, at pH5 (**a,d**), pH7 (**b,e**) and pH9 (**c,f**). In all cases, substrate (panels **a,b,c:** BHET or panels **d,e,f**: MHET) in 25 mM aqueous buffered solution at the specified pH and 10% DMSO. Source data are provided as a Source Data file.

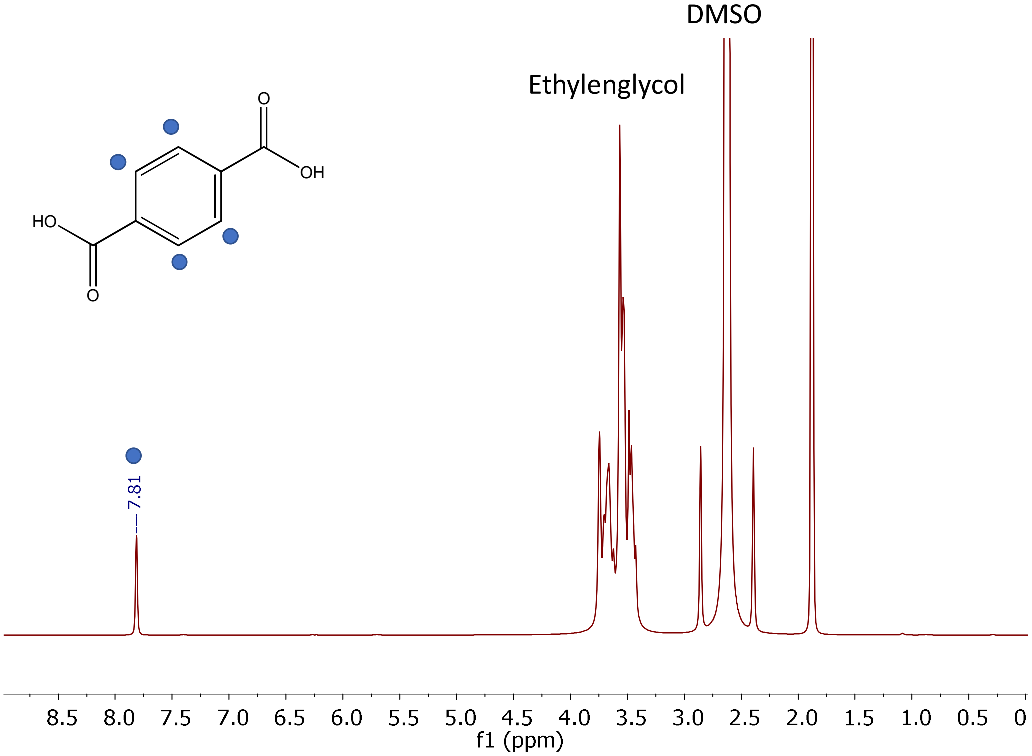

Supplementary Fig.6. ^{1}H NMR enzymatic reaction sample at pH 5 using crude BHET: (300 MHz, Deuterium Oxide, 298 K) δ 7.81 (S, 4H). Source data are provided as a Source Data file.

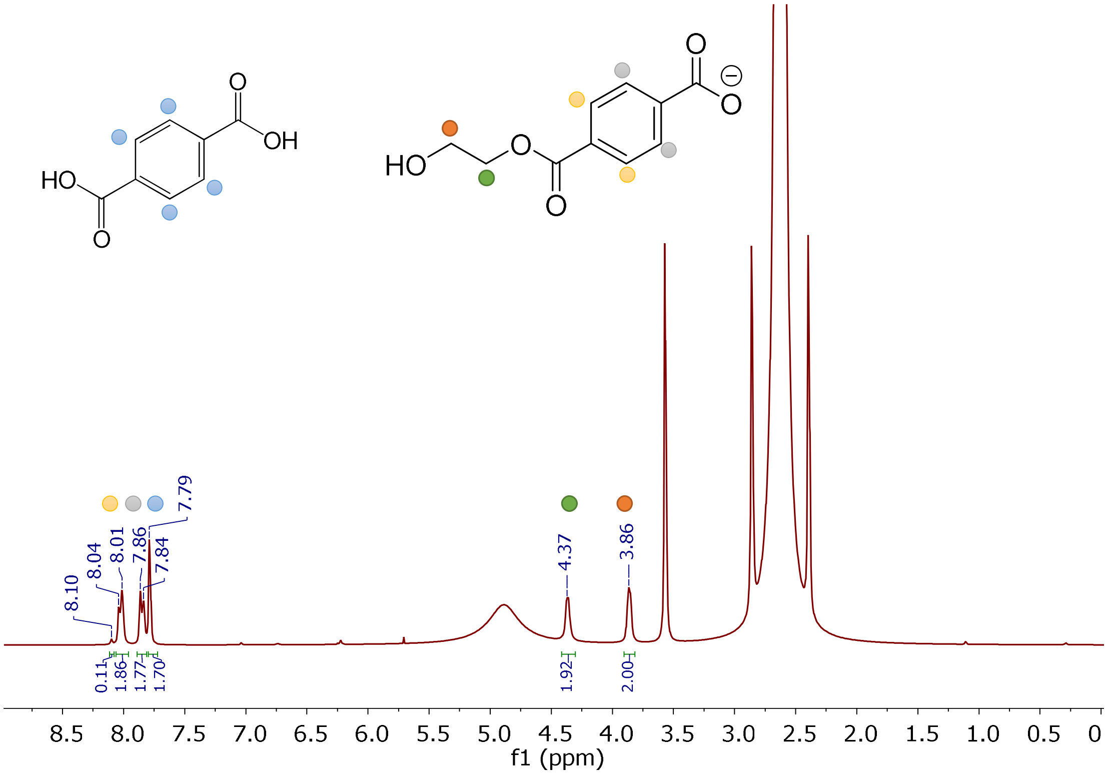

Supplementary Fig.7. ^{1}H NMR enzymatic reaction sample at pH 9 using crude BHET: (300 MHz, D_2O, 298 K) δ 8.03 (d, *J* = 8.5 Hz, 2H), 7.86 (d, *J* = 8.6 Hz, 2H), 7.78 (s, 9H), 4.36 (t, 2H), 3.86 (t, 2H). Source data are provided as a Source Data file.

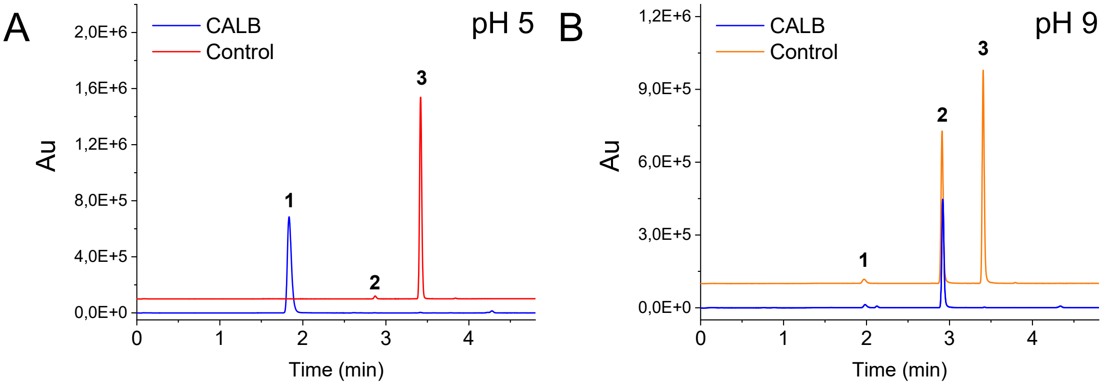

Supplementary Fig.8. UPLC-MS analysis of sample reaction at pH 5 or 9 from crude BHET. Reaction with enzyme (blue line), reaction control without enzyme (red and orange line). A) pH 5, B) pH 9. Retention peaks: 1, retained at 1.97 min corresponding to TPA; 2, retained at 2.92 min corresponding to MHET; 3, retained at 3.41 min corresponding to BHET. Source data are provided as a Source Data file.

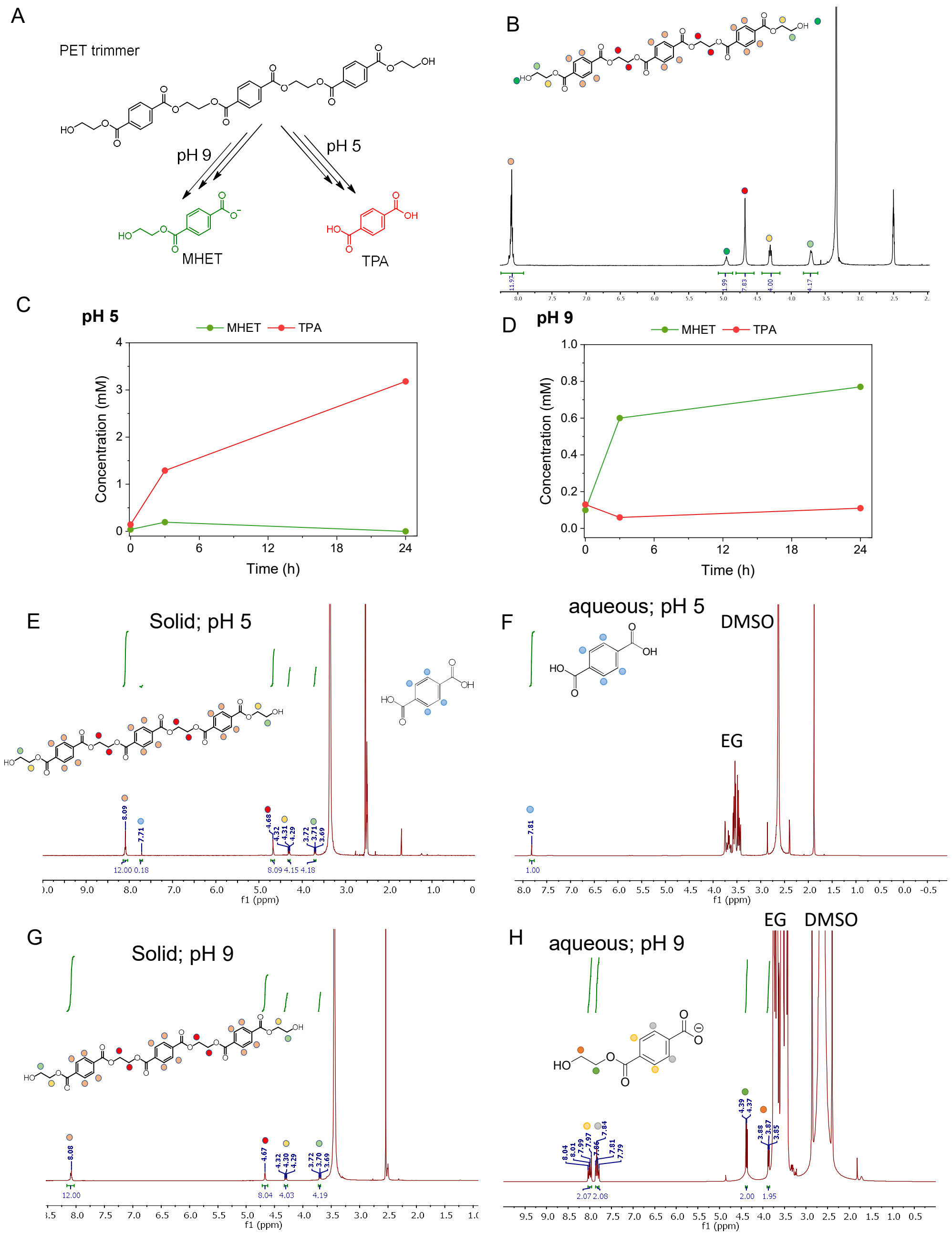

Supplementary Fig.9. Analysis of hydrolysis of a PET trimer at pH 5 or 9. The low solubility of the trimer in the reaction media led us to perform the reaction in a bi-phasic (solid-liquid) system. Upon 24 h reaction, precipitated (solid) and aqueous samples were withdrawn and analyzed by ^{1}H-NMR. Aqueous samples withdrawn at different times were also analyzed only by UPLC-MS. **A**) Reaction scheme. **B**) ^{1}H-NMR of the trimer before the enzymatic hydrolysis in pure DMSO. **C**) Reaction course analyzed by UPLC-MS at pH 5 and **D**) at pH 9. MHET (green line) and TPA (red line). Data are the mean value of n=2 independent measurements and error bars mean the standard deviation of the independent duplicates. **E**) ^{1}H-NMR of solid fractions solubilized in DMSO upon 24 hours reaction with CALB at pH 5, and **G**) at pH 9. **F**) ^{1}H-NMR aqueous fractions of the 24 hours reaction with CALB at pH 5, and **H**) at pH 9. EG: Ethylene glycol. DMSO: dimethylsulfoxide. Source data are provided as a Source Data file.

Supplementary Methods

Computational model set up. Wild type *Candida antarctica* Lipase B (CALB) initial geometry was taken from PDB structure 1TCA.[1] Systems were prepared where the protonation state of titratable residues was determined at pH equivalent to 5 and 9 using the semiempirical program PropKa ver. 3.0 3.[2] and by getting the full titratable curves based on constant-pH employing hybrid non-equilibrium molecular dynamic and Monte Carlo (neMD/MC) simulations.[3,4] as implemented as a Tcl plugin, namdcph, for use in conjunction with NAMD ver. 2.12.[5] All neMD/MC simulations were carried out with the CHARMM36 force field.[6] Constant-pH MD assays of the titration curves were performed on 55 pH values between 2.0 and 12.8 at intervals of 0.2 units and repeated five times. All simulations attempted protonation moves every 10 ps over 50 ns with switch times of 20 ps (i.e., 5000 neMD/MC cycles). The efficiency of sampling was improved by assigning inherent pKa values using the ones originally predicted by PropKa software. The protonation states of Lys, Glu, and Asp residues of CALB were explored during these simulations thus obtaining complete titratable curves that provide a robust estimation of the protonation state for selected residues. In the case of the system at pH 5, three protonated residues were taken into account, Asp134, Glu81, and Glu 284. In the case of pH 9 all former aspartic and glutamic residues, and Lys136 were deprotonated. The only histidine, the catalytic His224, was treated as neutral with a hydrogen atom added in Nδ position. Moreover, three disulphide bridges between residues Cys22 and Cys64, Cys216 and Cys258, and Cys293 and Cys311 were defined. The substrates BHET and MHET, in its neutral and negatively charged state, were manually built in the active site, with the oxygen of the carbonyl properly placed in the oxyanion hole formed by Thr40 and Gln106. Afterwards, all missing hydrogen atoms were added to the structure and the system was solvated into a $100 \times 80 \times 80$ Å^3 pre-equilibrated box of TIP3P water molecules. Water molecules with an oxygen atom within 2.8 Å of any heavy atom were removed. In the case of the system with a protonation state equivalent to that observed at pH 5, the charge of the full system was positive so one chlorine ion was added for its neutralization. In the case of pH 9, three sodium ions were inserted. Systems used in the simulations were composed of 64,664 atoms (4,627 belonging to the protein, 24 or 25 to the substrate, 1 or 2 ions and 60,012 to the water molecules) and 64,656 atoms (4,623 of the protein, 24 of the substrate, 3 ions and 60,006 belonging to the water molecules) for pH 5 and pH 9 respectively in the case of MHET. In the case of BHET, the system was made of 64,670 atoms (4,627 belonging to the protein, 32 to the substrate, 2 ions and 60,009 for the water molecules) and 64,666 atoms (4623 of the protein, 32 of the substrate, 2 ions and 60,009 belonging to the water molecules) for pH 5 and pH 9. Finally, the D134A CALB variant was prepared by replacing Asp134 by Ala, using Dicovery

Studio Visualizer v21.1.0.20298, and repeating all the setting up procedure described for the systems with the wild-type CALB.

After initial energy minimizations, the systems were heated to 303 K with 0.1 K temperature increment and equilibrated during short (100 ps) NPT MD simulations, followed by non-accelerated classical 100 ns NVT MD simulations with AMBER force field,[7] as implemented in NAMD software.[8] The missing force field parameters for the substrates were generated using GAFF[9] and the Antechamber[10] tool (Supplementary Table 1, 2 and 3). Partial charges were computed using the Austin Model 1 (AM1)[11] semiempirical Hamiltonian as implemented in Antechamber. During the 100 ns of NVT MD simulations, all atoms were free to move within periodic boundary conditions and cut-offs for nonbonding interactions with an internal cut-off of 14.5 Å and an external of 16 Å. In order to avoid diffusion of the substrate, a restraint in the position between the oxygen of the carbonyl and the oxyanion hole was applied. To maintain a constant temperature the Langevin thermostat[12] was applied.

The analysis of electrostatic and van der Waals interactions between the substrate and the protein during the MD was done using CPPTRAJ[13] as implemented in Ambertools.[14]

From MD simulations at the pH 5 and pH 9 with BHET and from the one at pH 5 with MHET, the most populated reactive structures were selected based on the distances of the reaction coordinates and were used to study the reaction mechanism using QM/MM MD simulations, as described below.

QM/MM simulations. In the present work, the standard additive hybrid QM/MM scheme was used to construct the total Hamiltonian, $\widehat{\mathrm{H}}_{\mathrm{QM/MM}}$, where the total energy $E_{QM/MM}$ is obtained as the sum of specific contributions, as presented in equation 1:

$$E_{QM/MM} = \langle \Psi | \widehat{H_o} | \Psi \rangle + \left(\sum \left\langle \Psi \left| \frac{q_{MM}}{r_{e,MM}} \right| \Psi \right\rangle + \sum \sum \frac{Z_{QM} q_{MM}}{r_{QM,MM}} \right) + E_{QM/MM}^{vdW} + E_{MM} \qquad (1)$$

where E_{MM} is the energy of the MM subsystem term, $E_{QM-MM}^{vdW}$the van der Waals interaction energy between the QM and MM subsystems and E_{QM-M}^{elect} includes both the Coulombic interaction of the QM nuclei (Z_{QM}) and the electrostatic interaction of the polarized electronic wave function (q_{MM}) with the charges of the protein (q_{MM}). The region described by quantum mechanics includes the side chains of the catalytic Ser105, His224 and Asp187 residues as well as the full substrate, and one water molecule, as shown in Supplementary Fig.9. Three-link atoms[60] were inserted where the QM/MM boundary intersected covalent bonds: these were placed between the Cα-Cβ for Ser105, His224 and Asp187. In the deacylation step, the leaving group was removed from the calculations.

The AM1 semiempirical Hamiltonian and the Minnesota Functional M06-2X,[15] with the standard 6-31+G(d,p) basis set, were used to treat the QM sub-set of atoms corresponding to the substrate and the catalytic residues of the active site as implemented in Mopac[16] and Gaussian 09,[17] respectively. A water molecule was also included in the QM region for the deacylation step. The OPLS-AA[18] and TIP3P[19] classical force fields were used to treat the protein and the solvent water molecules, respectively, as implemented in the fDynamo library.[20] The atom positions of all residues presented beyond 25 Å from the substrate were frozen and the same cut-offs as in MD simulations were applied for the nonbonding interactions. A first minimization of the full system was done using a combination of conjugate gradient and L-BFGS-B[21] algorithms implemented in the fDynamo library.

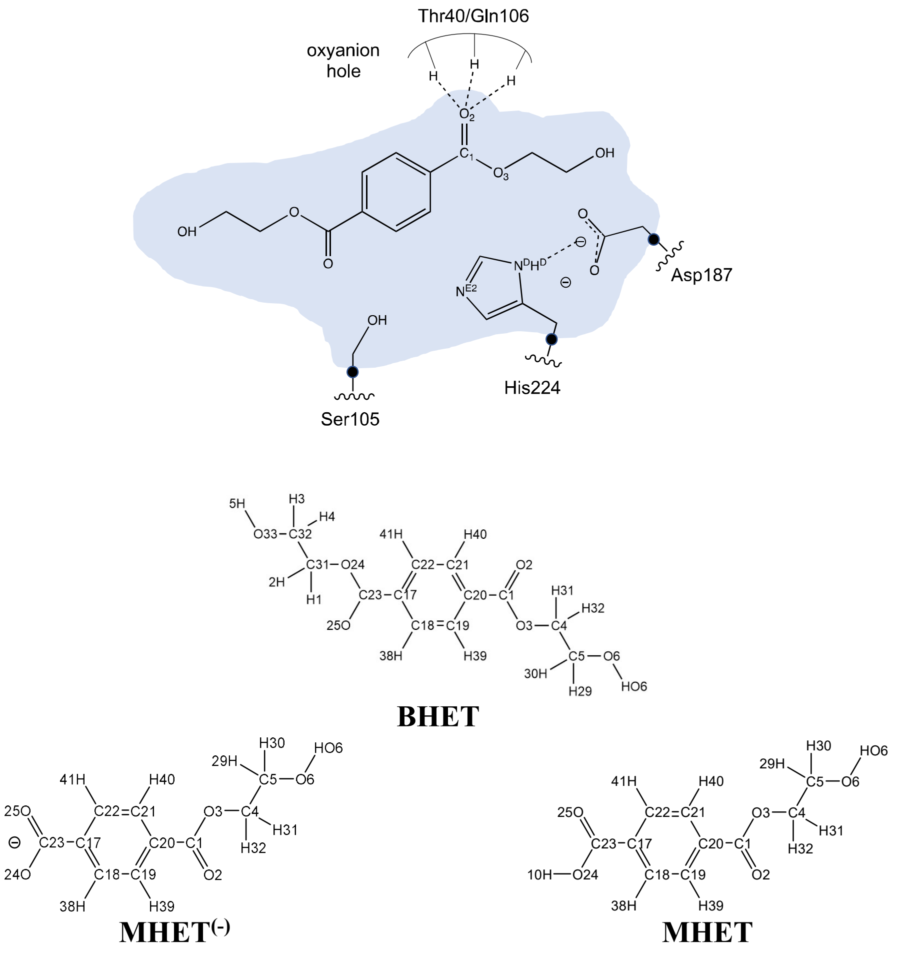

Supplementary Fig.10. Schematic representation of the QM sub-set region (shadowed region). Black dots represent link atoms between QM and MM regions. The H-bond interactions between the BHET substrate and the oxyanion hole, are shown as dashed lines (top panel). Numbering of the atoms in the BHET (middle panel) and in the MHET$^{(-)}$ and MHET (bottom panel).

Potential Energy Surfaces. Potential Energy Surfaces (PES) were explored by choosing and scanning the appropriate combination of internal coordinates (ξ_i) assuming their dominant role in the shape of the reaction coordinate. Thus, a combination of different distances was controlled during the exploration of all four chemical steps being part of the complete reaction path. In the first step of the reaction, the PES was generated by controlling the antisymmetric combination of the distance formed by OG and HG of Ser105, and the proton acceptor NE2 of His224, together with the forming of the bond between C1 of the substrate and OG of Ser105 were controlled, directing the acylation process. In the second step antisymmetric combination of nitrogen, NE2 atom and hydrogen HG, attached to His224 and this hydrogen atom and its acceptor, oxygen, O3 atom of the substrate, together with carbon-nitrogen (C1-O3) bond of a substrate were controlled. PES of the third step that starts the process of deacylation was generated by scanning the antisymmetric combination of distance between oxygen, O^{wat} atom of the water molecule and hydrogen, H^{wat} of the same molecule and the same hydrogen atom and nitrogen, NE2 atom of His224, together with a distance corresponding to the formation of the covalent bond between oxygen, O^{wat} from water and carbon, C1 atom of the substrate. The final, fourth step of the reaction is explored controlling the antisymmetric combination of distance between nitrogen, NE2 atom of His224 and hydrogen, H^{wat} atom and the same hydrogen atom and oxygen, OG atom of the Ser105, together with elongation distance between oxygen, OG atom of Ser105 and carbon, C1 atom of the substrate.

In order to explore all PESs, the harmonic constraint of 5000 $kJ \cdot mol^{-1} \cdot Å^{-2}$ was used to maintain the proper interatomic distances along the reaction coordinate, and a series of conjugate gradient optimizations and L-BFGS-B optimization algorithms were applied to obtain the final potential energy of the minimized constrained geometry. The QM sub-set of atoms was described by the AM1 semiempirical Hamiltonian. The distance evolution was controlled by applying a small size change of 0.1 Å when the distance between two heavy atoms was explored, or 0.05 Å when the transfer of light hydrogen atoms was involved.

A micro-macro iteration optimization algorithm[22,23] together with Baker's algorithm[24,25] was used to localize, optimize, and characterize the transition states (TS) and structures using a Hessian matrix containing all the coordinates of the QM subsystem, whereas the gradient norm of the remaining movable atoms was maintained less than 0.25 $kcal\ mol^{-1}\ Å^{-1}$. The Intrinsic Reaction Coordinate (IRC) was traced down from located TSs to the connecting valleys in mass-weighted Cartesian coordinates. And the same micro-macro iteration optimization algorithm was used to optimize reactant complex (RC), intermediates (Is) and product complex (PC). The existence of the saddle-points, as well as those located in minima, was confirmed by

frequency calculation. Thus, for TS structures, only one imaginary value of frequency was registered, while for structures located in the minimum of the PESs no imaginary values were found.

Free Energy Surfaces. FESs were obtained, in terms of two-dimensional potential mean force (2D-PMF),[26] for every step of the reaction using the Umbrella Sampling (US) approach[27],[28] combined with the Weighted Histogram Analysis Method (WHAM).[29] The procedure for the PMF calculation is straightforward and requires a series of molecular dynamics simulations in which the distinguished reaction coordinate variable, ξ, is constrained around particular values. The values of the variables sampled during the simulations are then pieced together to construct a distribution function from which the PMF is obtained as a function of the distinguished reaction coordinate (W(ξ)). The PMF is related to the normalized probability of finding the system at a particular value of the chosen coordinate by eq 2:

$$W(\xi) = C - kTln \int \rho(r^N)\delta(\xi(r^N) - \xi)dr^{N-1} \quad (2)$$

The activation free energy can be then expressed as:

$$\Delta G^{\ddagger}(\xi) = W(\xi^{\ddagger}) - \left[W(\xi^R) + G_{\xi}(\xi^R)\right] \quad (3)$$

where the superscripts indicate the value of the reaction coordinate at the reactants (R), and the TS (‡), and $G_{\xi}(\xi^R)$ is the free energy associated with setting the reaction coordinate to a specific value at the reactant state. Normally this last term makes a small contribution, and the activation free energy is directly estimated from the PMF change between the maximum of the profile and the reactant’s minimum:

$$\Delta G^{\ddagger}(\xi) \approx W(\xi^{\ddagger}) - W(\xi^R) = \Delta W^{\ddagger}(\xi) \quad (4)$$

The selection of the reaction coordinate is usually trivial when the mechanism can be driven by a single internal coordinate or a simple combination (as the antisymmetric combination of two interatomic distances). However, this is not the case for all possible steps of the reaction subject of study in this paper where many coordinates are participating. Instead, we were compelled to obtain a much more computationally demanding 2D-PMF using two coordinates: ξ_1 and ξ_2. The 2D-PMF is related to the probability of finding the system at particular values of these two coordinates:

$$W(\xi) = C' - kTln \int \rho(r^N)\delta(\xi_1(r^N) - \xi_1)\delta(\xi_2(r^N) - \xi_2)dr^{N-2} \quad (5)$$

To estimate the activation free energy from this quantity, we recovered one-dimensional PMF changes tracing a maximum probability reaction path on the 2D-PMF surface and integrating over the perpendicular coordinate.

Thus, a series of MD simulations were performed adding a constraint for the selected reaction coordinates with an umbrella force constant of 2500 $kJ \cdot mol^{-1} \cdot Å^{-2}$. In every window, QM/MM MD simulations were performed with a total of 5 ps of equilibration and 20 ps of production at 303 K using the Langevin-Verlet algorithm[30] with a time step of 1 fs. Structures obtained in previously computed PESs were used as starting points for the MD simulations in every window.

Spline corrections. In order to improve lower quality results associated with the low-level semiempirical calculations, high-level corrections were applied using Density Functional Theory (DFT). As already described in the literature,[31,32] a correction term $S[\Delta E_{LL}^{HL}(\xi_1, \xi_2)]$ is interpolated to any value along reaction coordinates in the FES. A continuous energy function is used to obtain the corrected PMFs:

$$E = E_{LL/MM} + S[\Delta E_{LL}^{HL}(\xi_1, \xi_2)] \quad (6)$$

where S is the two-dimensional spline function and ΔE_{LL}^{HL} is the difference between the energies obtained at low-level (LL) and high-level (HL) of the theory of the QM part. In this work the AM1 semiempirical Hamiltonian was used as the LL method, while the DFT method was selected for the HL energy calculation. In particular, HL energy calculations were performed by means of the hybrid M06-2X functional using the standard 6-31+G(d,p) basis set. These calculations were carried out using the Gaussian09 program.

Supplementary Table 1. Atom types, charges (in a.u.) and parameters obtained after parametrization of BHET obtained using structure molecule structure optimized at AM1 level of theory and GAFF force field. See Supplementary Fig.10 for representation of the atom numbering.

Atom name	Atom type	Charge	Atom name	Atom type	Charge
O6	oh	-0.5988	C19	ca	-0.09025
HO6	ho	0.4055	H39	ha	0.1635
C5	c3	0.1124	C18	ca	-0.09025
H29	h1	0.0402	H38	ha	0.1635
H30	h1	0.0402	C17	ca	-0.1026
C4	c3	0.1379	C23	c	0.6437
H31	h1	0.0742	O25	o	-0.5365
H32	h1	0.0742	O24	os	-0.4374
O3	os	-0.4374	C31	c3	0.1379
C1	c	0.6437	H1	h1	0.0742
O2	o	-0.5365	H2	h1	0.0742
C20	ca	-0.1026	C32	c3	0.1124
C21	ca	-0.09025	H3	h1	0.0402
C22	ca	-0.09025	H4	h1	0.0402
H41	ha	0.1635	O33	oh	-0.5988
H40	ha	0.1635	H5	ho	0.4055

**MASS**			**DIHEDRAL**				
oh	16.000	0.465	h1-c3-c3-oh	1	0.000	0.000	-3.000
ho	1.008	0.135	h1-c3-c3-oh	1	0.250	0.000	1.000
c3	12.010	0.878	oh-c3-c3-os	1	0.144	0.000	-3.000
h1	1.008	0.135	oh-c3-c3-os	1	1.175	0.000	2.000
os	16.000	0.465	h1-c3-oh-ho	3	0.500	0.000	3.000
c	12.010	0.616	c3-c3-oh-ho	1	0.160	0.000	-3.000
o	16.000	0.434	c3-c3-oh-ho	1	0.250	0.000	1.000
ca	12.010	0.360	c3-c3-os-c	1	0.383	0.000	-3.000
ha	1.008	0.135	c3-c3-os-c	1	0.800	180.000	1.000
**BOND**			h1-c3-c3-h1	9	1.400	0.000	3.000
ho-oh	371.40	0.973	h1-c3-c3-os	1	0.000	0.000	-3.000
c3-oh	316.70	1.423	h1-c3-c3-os	1	0.250	0.000	1.000
c3-h1	330.60	1.097	o -c -os-c3	1	2.700	180.000	-2.000
c3-c3	300.90	1.538	o -c -os-c3	1	1.400	180.000	1.000
c3-os	308.60	1.432	ca-c -os-c3	2	5.400	180.000	2.000
c -os	390.80	1.358	h1-c3-os-c	3	1.150	0.000	3.000
c -o	637.70	1.218	os-c -ca-ca	4	4.000	180.000	2.000
c -ca	345.90	1.491	c -ca-ca-ca	4	14.500	180.000	2.000
ca-ca	461.10	1.398	c -ca-ca-ha	4	14.500	180.000	2.000
ca-ha	345.80	1.086	o -c -ca-ca	4	4.000	180.000	2.000
**ANGLE**			ca-ca-ca-ha	4	14.500	180.000	2.000
h1-c3-oh	50.900	110.260	ca-ca-ca-ca	4	14.500	180.000	2.000
c3-c3-oh	67.500	110.190	ha-ca-ca-ha	4	14.500	180.000	2.000
c3-oh-ho	47.400	107.260	**IMPROPER**				
c3-c3-h1	46.400	109.560	ca-o -c -os	1.1	180.0	2.0	
c3-c3-os	68.000	107.970	c -ca-ca-ca	1.1	180.0	2.0	
h1-c3-h1	39.200	108.460	ca-ca-ca-ha	1.1	180.0	2.0	
c -os-c3	63.300	115.980	**NONBON**				
h1-c3-os	50.800	109.780	oh	1.7210	0.2104		
o -c -os	75.300	123.250	ho	0.0000	0.0000		
ca-c -os	69.300	112.440	c3	1.9080	0.1094		
c -ca-ca	64.300	120.330	h1	1.3870	0.0157		
ca-c -o	68.700	122.600	os	1.6837	0.1700		
ca-ca-ca	66.600	120.020	c	1.9080	0.0860		
ca-ca-ha	48.200	119.880	o	1.6612	0.2100		
			ca	1.9080	0.0860		
			ha	1.4590	0.0150		

Supplementary Table 2. Atom types, charges (in a.u.) and parameters obtained after parametrization of MHET$^{(-)}$, obtained using structure molecule structure optimized at AM1 level of theory and GAFF force field.

Atom name	Atom type	Charge	Atom name	Atom type	Charge
O6	oh	-0.6198	C19	ca	-0.0935
HO6	ho	0.4310	H39	ha	0.1295
C5	c3	0.1114	C18	ca	-0.1325
H29	h1	0.0472	H38	ha	0.1605
H30	h1	0.0472	C17	ca	-0.0796
C4	c3	0.1014	C23	c	0.9042
H31	h1	0.0632	O24	o	-0.8198
H32	h1	0.0632	O25	o	-0.8198
O3	os	-0.4439	C22	ca	-0.1325
C1	c	0.6667	H41	ha	0.1605
O2	o	-0.6000	C21	ca	-0.0935
C20	ca	-0.1806	H40	ha	0.1295

**MASS**			**DIHEDRAL**				
oh	16.000	0.465	h1-c3-c3-oh	1	0.000	0	-3
ho	1.008	0.135	h1-c3-c3-oh	1	0.250	0	1
c3	12.010	0.878	oh-c3-c3-os	1	0.144	0	-3
h1	1.008	0.135	oh-c3-c3-os	1	1.175	0	2
os	16.000	0.465	h1-c3-oh-ho	3	0.500	0	3
c	12.010	0.616	c3-c3-oh-ho	1	0.160	0	-3
o	16.000	0.434	c3-c3-oh-ho	1	0.250	0	1
ca	12.010	0.360	c3-c3-os-c	1	0.383	0	-3
ha	1.008	0.135	c3-c3-os-c	1	0.800	180	1
**BOND**			h1-c3-c3-h1	9	1.400	0	3
ho-oh	371.400	0.973	h1-c3-c3-os	1	0.000	0	-3
c3-oh	316.700	1.423	h1-c3-c3-os	1	0.250	0	1
c3-h1	330.600	1.097	o-c-os-c3	1	2.700	180	-2
c3-c3	300.900	1.538	o-c-os-c3	1	1.400	180	1
c3-os	308.600	1.432	ca-c-os-c3	2	5.400	180	2
c-os	390.800	1.358	h1-c3-os-c	3	1.150	0	3
c-o	637.700	1.218	os-c-ca-ca	4	4.000	180	2
c-ca	345.900	1.491	c-ca-ca-ha	4	14.500	180	2
ca-ca	461.100	1.398	c-ca-ca-ca	4	14.500	180	2
ca-ha	345.800	1.086	o-c-ca-ca	4	4.000	180	2
**ANGLE**			ca-ca-ca-ha	4	14.500	180	2
h1-c3-oh	50.90	110.26	ca-ca-ca-ca	4	14.500	180	2
c3-c3-oh	67.50	110.19	ha-ca-ca-ha	4	14.500	180	2
c3-oh-ho	47.40	107.26	**IMPROPER**				
c3-c3-h1	46.40	109.56	ca-o-c-os	1.1	180	2	
c3-c3-os	68.00	107.97	c-ca-ca-ca	1.1	180	2	
h1-c3-h1	39.20	108.46	ca-ca-ca-ha	1.1	180	2	
c-os-c3	63.30	115.98	ca-o-c-o	1.1	180	2	
h1-c3-os	50.80	109.78	**NONBON**				
o-c-os	75.30	123.25	oh	1.7210	0.2104		
ca-c-os	69.30	112.44	ho	0.0000	0.0000		
c-ca-ca	64.30	120.33	c3	1.9080	0.1094		
ca-c-o	68.70	122.60	h1	1.3870	0.0157		
ca-ca-ha	48.20	119.88	os	1.6837	0.1700		
ca-ca-ca	66.60	120.02	c	1.9080	0.0860		
o-c-o	77.90	130.25	o	1.6612	0.2100		
			ca	1.9080	0.0860		
			ha	1.4590	0.0150		

Supplementary Table 3. Atom types, charges (in a.u.) and parameters obtained after parametrization of MHET, obtained using structure molecule structure optimized at AM1 level of theory and GAFF force field.

Atom name	Atom type	Charge	Atom name	Atom type	Charge
O6	oh	-0.5988	C21	ca	-0.0785
HO6	ho	0.4080	H40	ha	0.1660
C5	c3	0.0994	C22	ca	-0.1050
H29	h1	0.0402	H41	ha	0.1545
H30	h1	0.0402	C17	ca	-0.1426
C4	c3	0.1384	C23	c	0.6357
H32	h1	0.0797	O24	oh	-0.5741
H31	h1	0.0797	H10	ho	0.4280
O3	os	-0.4349	O25	o	-0.4750
C1	c	0.6417	C18	ca	-0.1050
O2	o	-0.5350	H38	ha	0.1545
C20	ca	-0.1076	C19	ca	-0.0785
			H39	ha	0.1660

**MASS**			**DIHEDRAL**				
oh	16.000	0.465	h1-c3-c3-oh	1	0.000	0.000	-3.000
ho	1.008	0.135	h1-c3-c3-oh	1	0.250	0.000	1.000
c3	12.010	0.878	oh-c3-c3-os	1	0.144	0.000	-3.000
h1	1.008	0.135	oh-c3-c3-os	1	1.175	0.000	2.000
os	16.000	0.465	h1-c3-oh-ho	3	0.500	0.000	3.000
c	12.010	0.616	c3-c3-oh-ho	1	0.160	0.000	-3.000
o	16.000	0.434	c3-c3-oh-ho	1	0.250	0.000	1.000
ca	12.010	0.360	c3-c3-os-c	1	0.383	0.000	-3.000
ha	1.008	0.135	c3-c3-os-c	1	0.800	180.000	1.000
**BOND**			h1-c3-c3-h1	9	1.400	0.000	3.000
ho-oh	371.40	0.973	h1-c3-c3-os	1	0.000	0.000	-3.000
c3-oh	316.70	1.423	h1-c3-c3-os	1	0.250	0.000	1.000
c3-h1	330.60	1.097	o -c -os-c3	1	2.700	180.000	-2.000
c3-c3	300.90	1.538	o -c -os-c3	1	1.400	180.000	1.000
c3-os	308.60	1.432	ca-c -os-c3	2	5.400	180.000	2.000
c -os	390.80	1.358	h1-c3-os-c	3	1.150	0.000	3.000
c -o	637.70	1.218	os-c -ca-ca	4	4.000	180.000	2.000
c -ca	345.90	1.491	c -ca-ca-ha	4	14.500	180.000	2.000
ca-ca	461.10	1.398	c -ca-ca-ca	4	14.500	180.000	2.000
ca-ha	345.80	1.086	o -c -ca-ca	4	4.000	180.000	2.000
c -oh	400.10	1.351	ca-ca-ca-ha	4	14.500	180.000	2.000
**ANGLE**			ca-ca-ca-ca	4	14.500	180.000	2.000
h1-c3-oh	50.900	110.260	ha-ca-ca-ha	4	14.500	180.000	2.000
c3-c3-oh	67.500	110.190	oh-c -ca-ca	4	4.000	180.000	2.000
c3-oh-ho	47.400	107.260	ca-c -oh-ho	2	4.600	180.000	2.000
c3-c3-h1	46.400	109.560	o -c -oh-ho	1	2.300	180.000	-2.000
c3-c3-os	68.000	107.970	o -c -oh-ho	1	1.900	0.000	1.000
h1-c3-h1	39.200	108.460	**IMPROPER**				
c -os-c3	63.300	115.980	ca-o -c -os		1.1	180.0	2.0
h1-c3-os	50.800	109.780	c -ca-ca-ca		1.1	180.0	2.0
o -c -os	75.300	123.250	ca-ca-ca-ha		1.1	180.0	2.0
ca-c -os	69.300	112.440	ca-o -c -oh		1.1	180.0	2.0
c -ca-ca	64.300	120.330	**NONBON**				
ca-c -o	68.700	122.600	oh	1.7210	0.2104		
ca-ca-ha	48.200	119.880	ho	0.0000	0.0000		
ca-ca-ca	66.600	120.020	c3	1.9080	0.1094		
ca-c -oh	69.200	113.450	h1	1.3870	0.0157		
c -oh-ho	49.900	106.550	os	1.6837	0.1700		
o -c -oh	75.900	122.100	c	1.9080	0.0860		
			o	1.6612	0.2100		
			ca	1.9080	0.0860		
			ha	1.4590	0.0150		

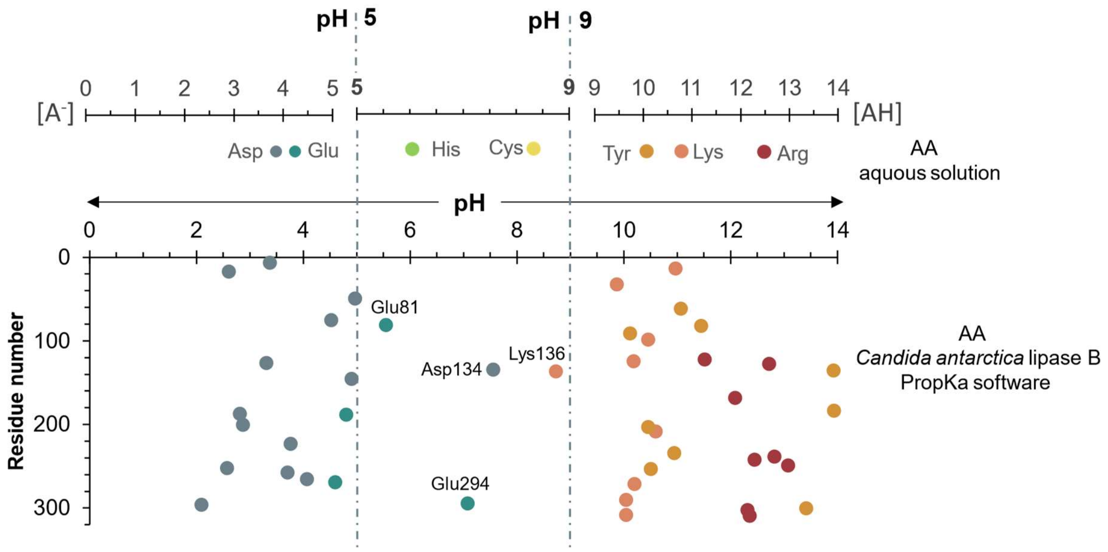

Supplementary Fig 11. pKa values of the titratable residues of CALB as derived from the PropKa ver. 3.0 3. Source data are provided as a Source Data file.

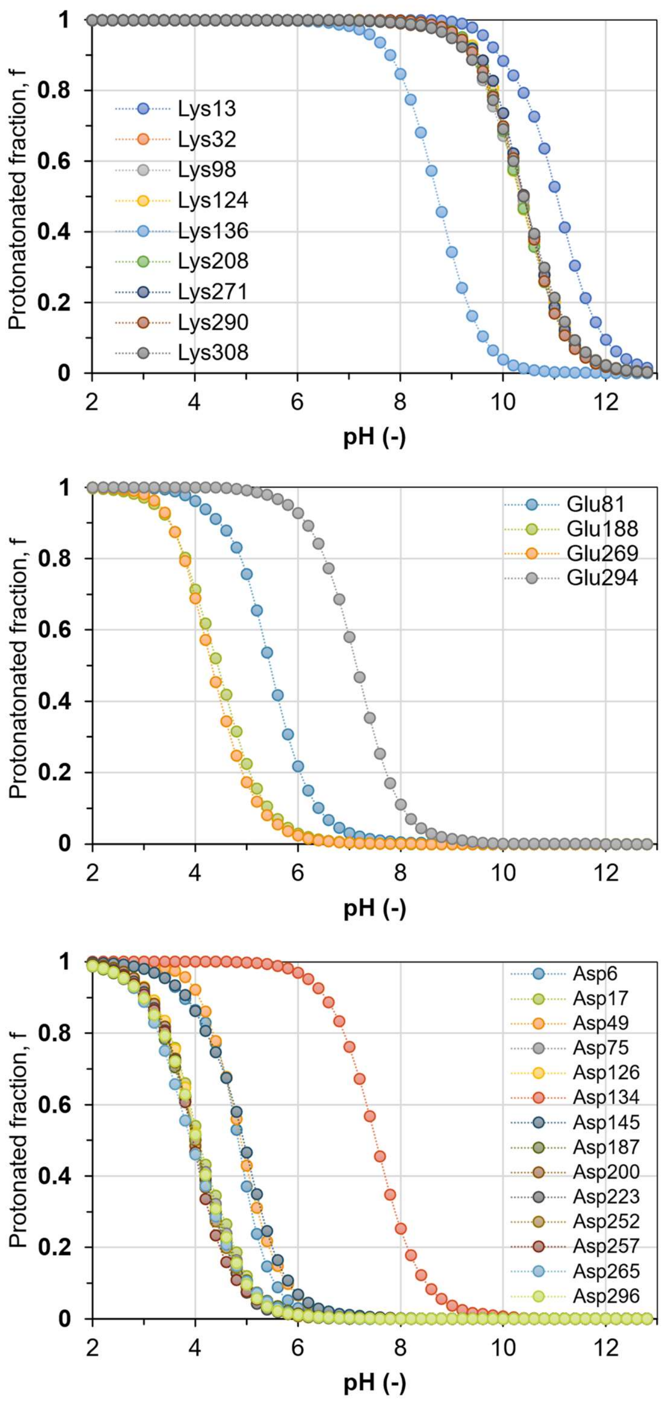

Supplementary Fig.12. Titratable curves for Lys residues (top panel), Glu residues (center panel) and Asp residues (bottom panel) of CALB, as obtained by means of the pH constant MD simulations. Source data are provided as a Source Data file.

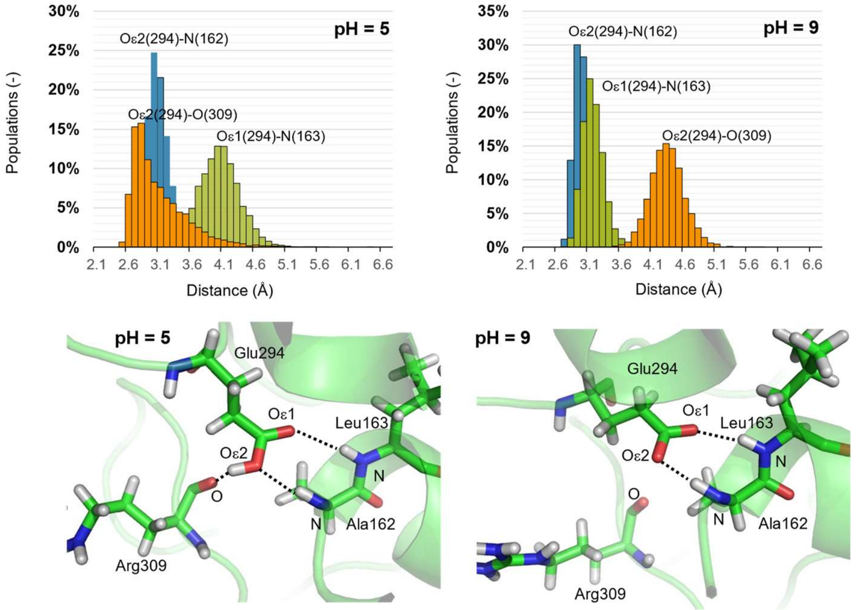

Supplementary Fig.13. Geometrical analysis of the pattern of interactions established between **Glu294** and its surroundings at pH 5 and pH 9. Source data are provided as a Source Data file.

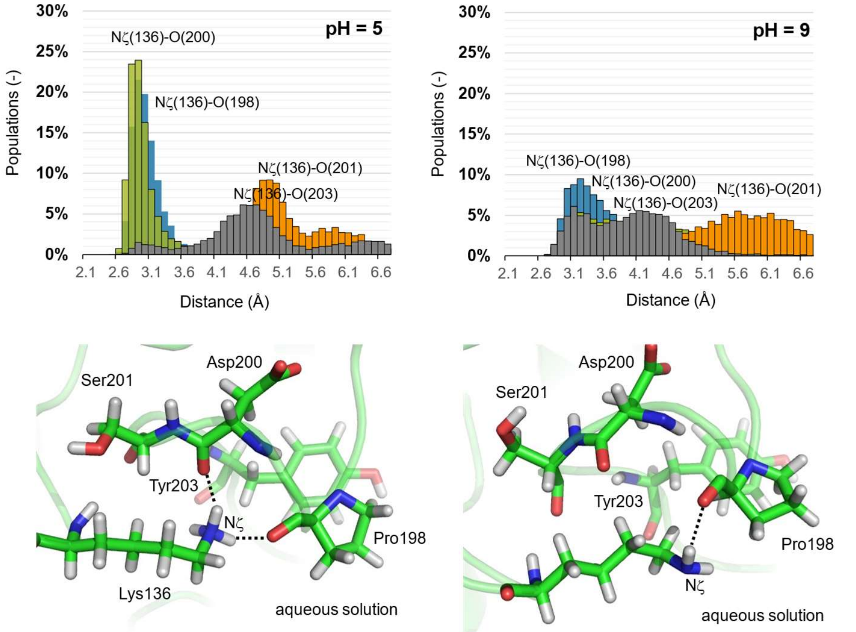

Supplementary Fig.14. Geometrical analysis of the pattern of interactions established between **Lys136** and its surroundings at pH 5 and pH 9. Source data are provided as a Source Data file.

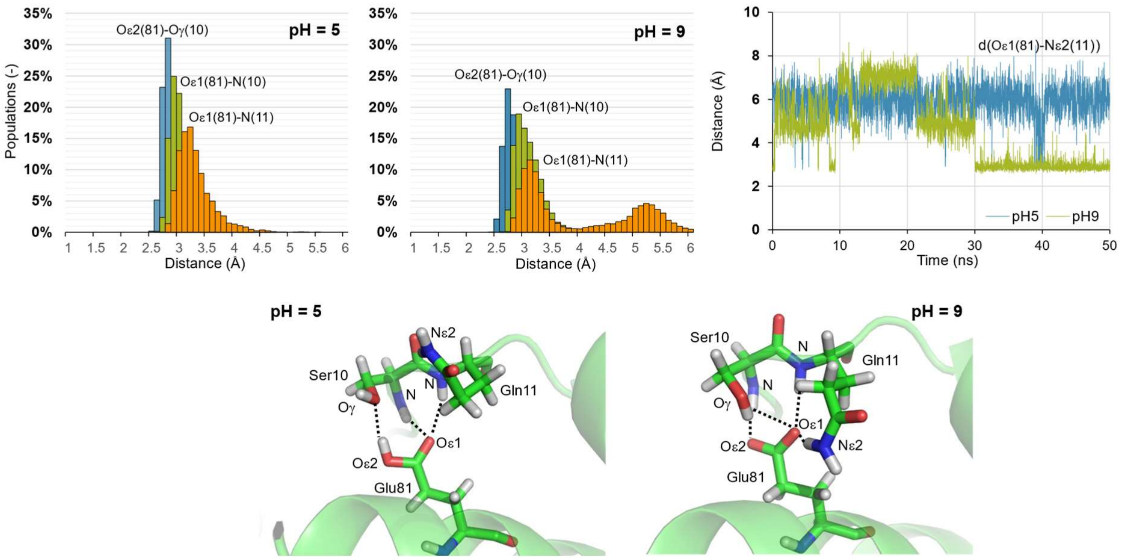

Supplementary Fig.15. Geometrical analysis of the pattern of interactions established between Glu81 and its surroundings at pH 5 and pH 9. Source data are provided as a Source Data file.

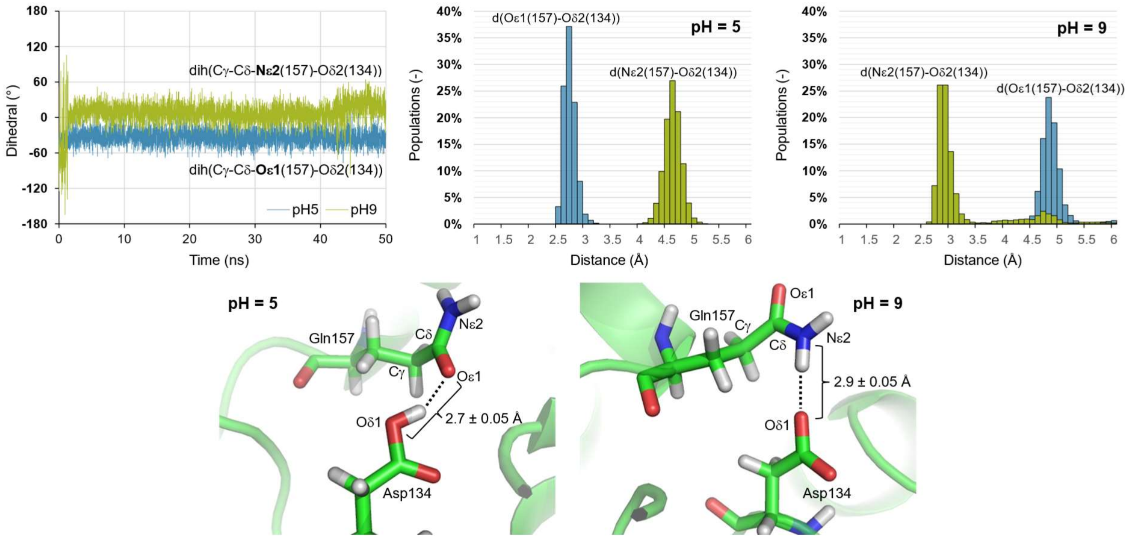

Supplementary Fig.16. Geometrical analysis of the pattern of interactions established between Gln157 and its surroundings at pH 5 and pH 9. Source data are provided as a Source Data file.

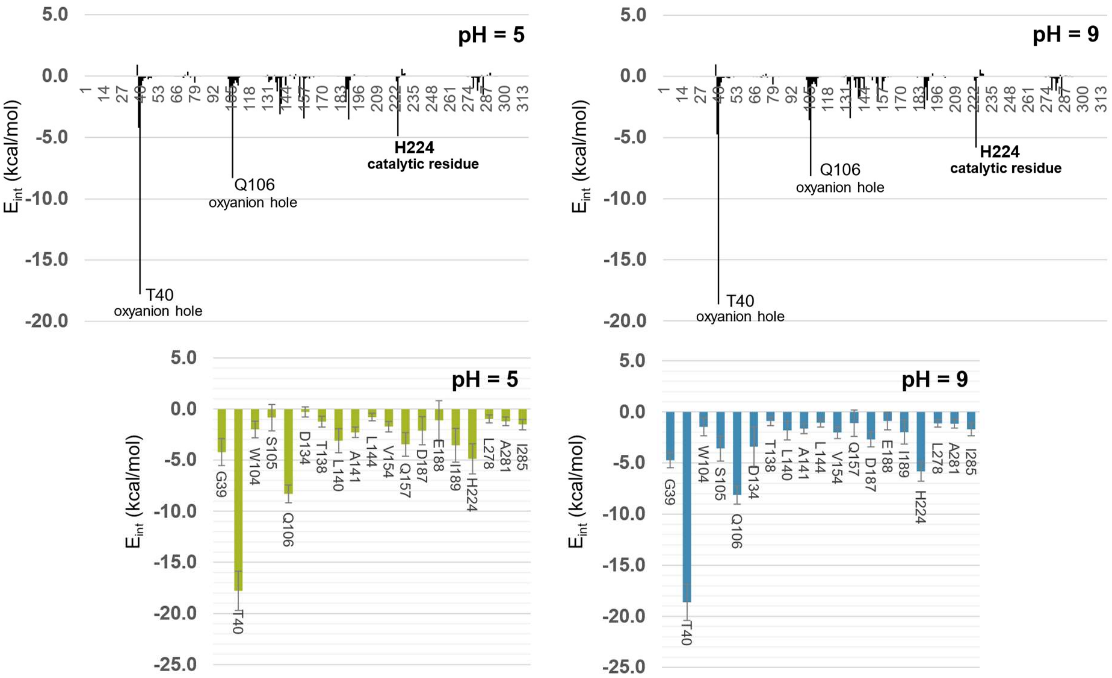

Supplementary Fig.17. Interactions between BHET and CALB, decomposed per residue, obtained over 1000 snapshots generated during the 100 ns of classical MD simulations at pH 5 and at pH 9 (n = 1000). Lower panels show those interactions with values larger than 1 kcal·mol-1, including standard deviations as error bars. Source data are provided as a Source Data file.

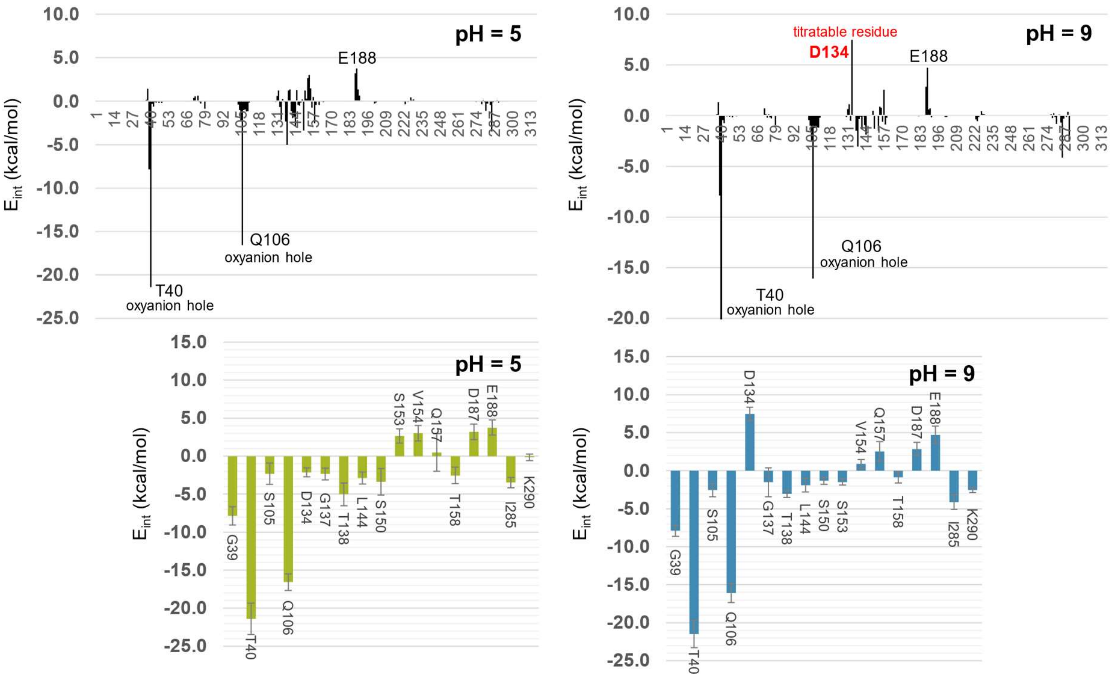

Supplementary Fig.18. Interactions between MHET$^{(-)}$ and CALB, decomposed per residue, obtained over 1000 snapshots generated during the 100 ns of classical MD simulations at pH 5 and at pH 9 (n = 1000). Lower panels show those interactions with values larger than 1 kcal·mol-1, including standard deviations as error bars. Source data are provided as a Source Data file.

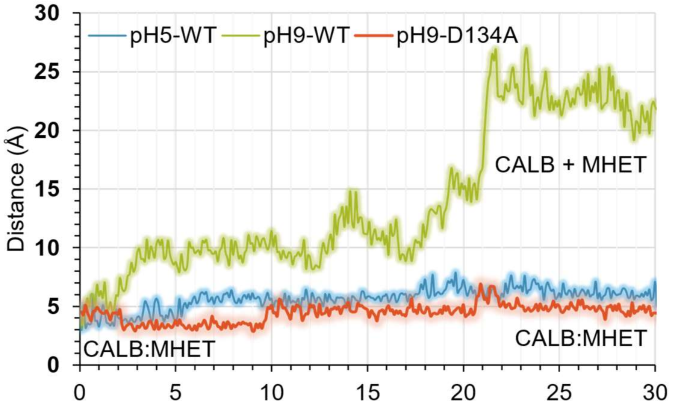

Supplementary Fig.19. Stability of CALB:MHET complex systems in WT and D134A variant. Time dependence evolution of the distance between the centre of mass of the substrate and the oxyanion hole along the unconstraint MD simulation in the CALB:MHET reactants complex at pH 5 (blue line) and at pH 9 of wild type (green line) and D134A variant (red line). Source data are provided as a Source Data file.

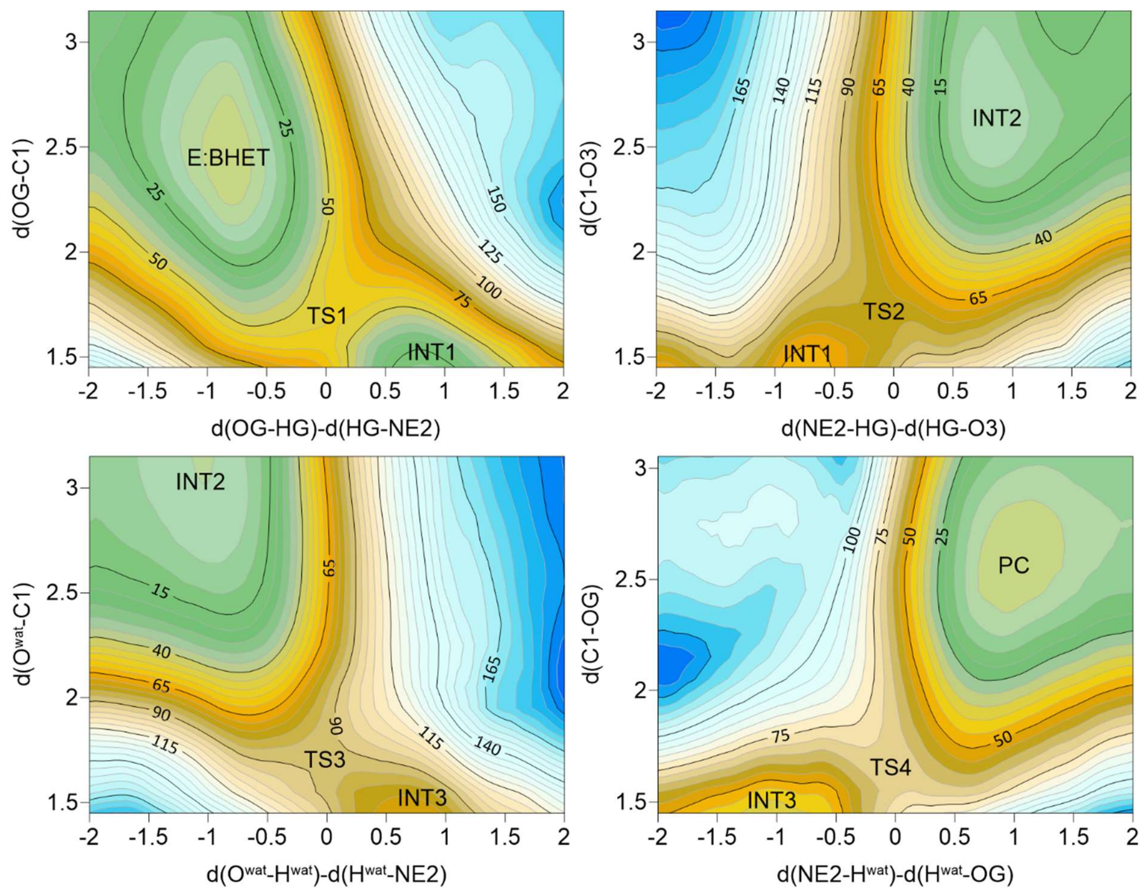

Supplementary Fig.20. M06-2X:AM1/MM free energy surfaces corresponding to the hydrolysis of BHET catalysed by CALB at pH 5. Values of isoenergetic lines are in kJ·mol^{-1} while values in axis are in Å. Source data are provided as a Source Data file.

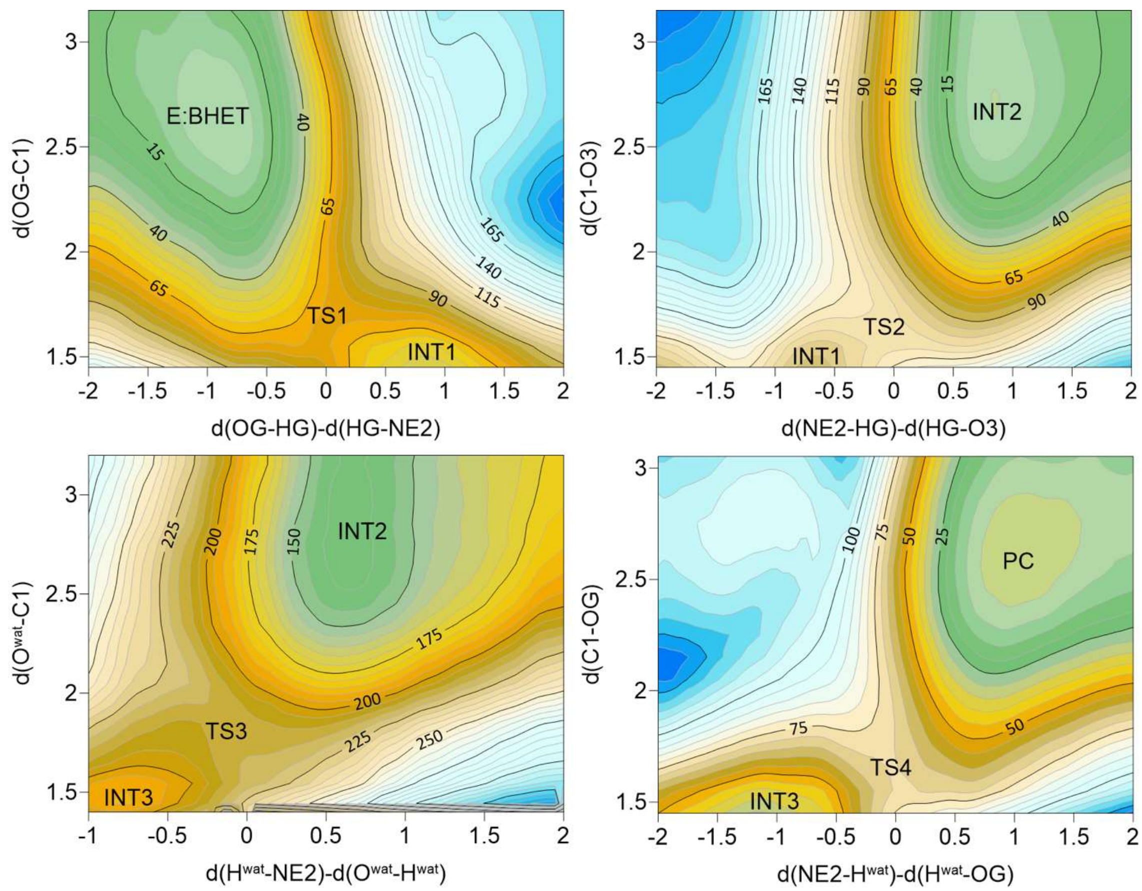

Supplementary Fig.21. M06-2X:AM1/MM free energy surfaces corresponding to the hydrolysis of BHET catalysed by CALB at pH 9. Values of isoenergetic lines are in kJ·mol^{-1} while values in axis are in Å. Source data are provided as a Source Data file.

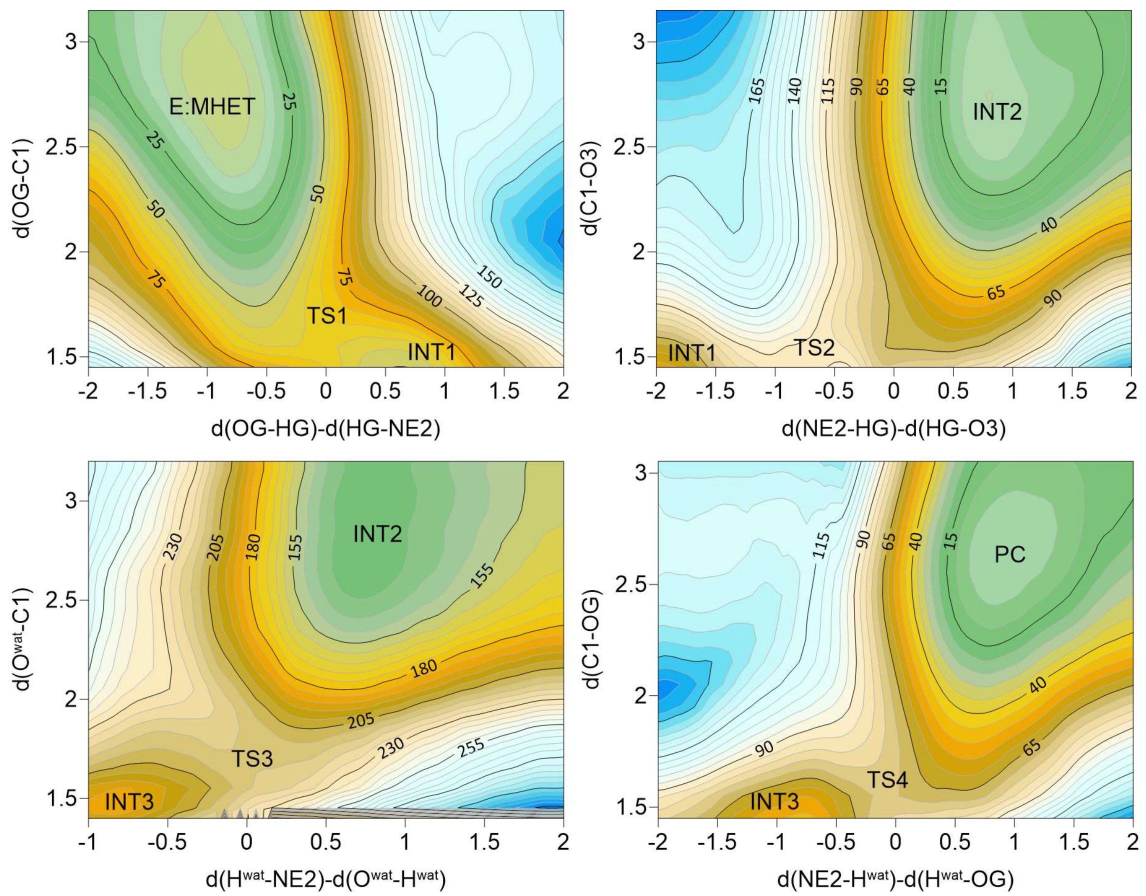

Supplementary Fig.22. M06-2X:AM1/MM free energy surfaces corresponding to the hydrolysis of MHET catalysed by CALB at pH 5. Values of isoenergetic lines are in kJ·mol^{-1} while values in axis are in Å. Source data are provided as a Source Data file.

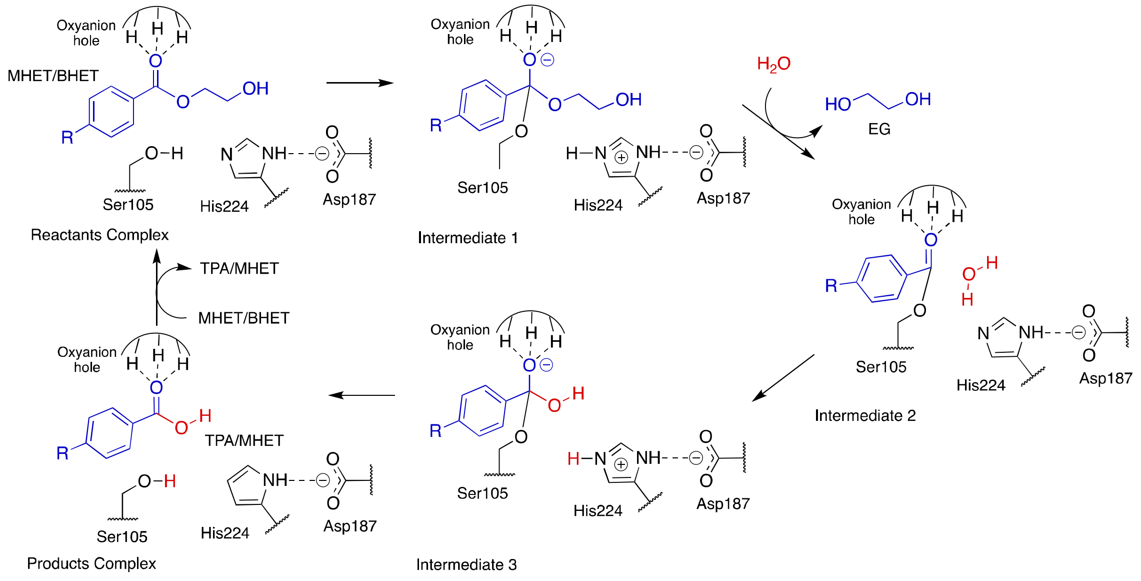

Supplementary Fig.23. Schematic representation of the reaction mechanism of the hydrolysis of the BHET/MHET catalysed by CALB. Acylation step: the nucleophilic attack of Ser105 to the carbonyl followed by the breaking of the C-O bond is triggered by His224, rendering the acyl-enzyme complex and EG. Hydrolysis step: the nucleophilic attack of a water molecule followed by the resolution of the acyl-enzyme complex is triggered again by His224, yielding TPA as a product. For BHET R=$COOCH_2CH_2OH$ while for MHET R=COO^-.

Supplementary Table 4. M06-2X:AM1/MM relative free energies of the states involved in the hydrolysis of $MHET^{(-)}$ catalysed by CALB at pH 5, and BHET catalysed by CALB at pH 5 and pH 9, derived from the FESs displayed in Figs S20, S21 and 22, and after including zero point vibrational energies corrections from structures optimized at M06-2X/MM level (PMF+ZPE). All values are reported in $kcal \cdot mol^{-1}$.

PMF

	RC	TS1	I1	TS2	I2	TS3	I3	TS4	PC
BHET pH 5	0.0	13.2	4.9	8.7	-9.7	7.3	2.2	9.7	-6.5
MHET pH 5	0.0	14.5	12.0	21.1	-5.8	12.7	6.2	11.3	-5.4
BHET pH 9	0.0	17.0	11.0	14.6	-11.2	6.9	1.3	10.5	-6.1

PMF + ZPE

	RC	TS1	I1	TS2	I2	TS3	I3	TS4	PC
BHET pH 5	0.0	9.3	4.5	6.9	-9.7	5.3	3.2	7.4	-4.8
MHET pH 5	0.0	11.1	11.0	18.3	-6.4	9.9	5.9	8.4	-5.1
BHET pH 9	0.0	13.2	10.1	13.4	-11.5	4.1	0.3	6.5	-7.1

Supplementary Table 5. Key inter-atomic distances (in Å) of structures optimized at M06-2X:AM1/MM level for the hydrolysis of BHET at pH 5 and pH 9, and for the hydrolysis of MHET at pH 5.

hydrolysis of BHET at pH 5

	RC	TS1	INT1	INT1	TS2	INT2	INT2	TS3	INT3	INT3	TS4	PC
$OG_{Ser105} – C1_{subs}$	2.48	2.08	1.46	1.43	1.40	1.44	1.32	1.43	1.44	1.48	1.78	2.36
$OG_{Ser105} – HG_{Ser105}$	0.98	1.59	2.56	2.85	2.81	2.83	-	-	-	-	-	-
$NE2_{His224} – HG_{Ser105}$	2.18	1.17	1.02	1.06	1.11	1.75	-	-	-	-	-	-
$C1_{subs} – O3_{subs}$	1.32	1.36	1.46	1.57	1.72	2.62	-	-	-	-	-	-
$HG_{Ser105} – O3_{subs}$	3.05	3.29	3.41	1.72	1.47	0.99	-	-	-	-	-	-
$O_{wat} – C1_{subs}$	-	-	-	-	-	-	2.74	1.75	1.49	1.46	1.40	1.34
$O_{wat} – H1_{wat}$	-	-	-	-	-	-	0.99	1.29	1.90	3.03	2.80	2.81
$H1_{wat} – NE2_{His224}$	-	-	-	-	-	-	1.87	1.24	1.04	1.03	1.28	2.11
$H1_{wat} – OG_{Ser105}$	-	-	-	-	-	-	3.01	2.94	3.03	2.36	1.38	0.98
$HD_{His224} – OD2_{Asp187}$	1.71	1.67	1.56	1.73	1.80	1.79	1.81	1.78	1.68	1.57	1.74	1.74
$HD_{His224} – ND_{His224}$	1.04	1.05	1.07	1.05	1.04	1.03	1.03	1.04	1.06	1.08	1.04	1.04
$C1_{subs} – O2_{subs}$	1.23	1.24	1.30	1.29	1.27	1.23	1.22	1.27	1.30	1.30	1.26	1.22
$O2_{subs} – H_{Thr40}$	1.89	2.01	1.82	1.72	1.74	1.80	1.82	1.73	1.70	1.77	1.89	1.74
$O2_{subs} – HG1_{Thr40}$	1.71	1.85	1.61	2.17	2.21	1.94	1.68	1.87	1.74	1.70	1.85	1.96
$O2_{subs} – H_{Gln106}$	1.90	1.84	1.67	2.47	2.47	2.07	2.21	2.60	2.44	1.81	1.88	1.96

hydrolysis of BHET at pH 9

	RC	TS1	INT1	INT1	TS2	INT2	INT2	TS3	INT3	INT3	TS4	PC
$OG_{Ser105} – C1_{subs}$	2.25	1.66	1.47	1.46	1.44	1.32	1.31	1.40	1.42	1.46	1.64	2.49
$OG_{Ser105} – HG_{Ser105}$	0.98	1.37	2.08	2.49	2.74	2.85	-	-	-	-	-	-
$NE2_{His224} – HG_{Ser105}$	1.99	1.28	1.04	1.03	1.03	1.93	-	-	-	-	-	-
$C1_{subs} – O3_{subs}$	1.34	1.42	1.47	1.48	1.54	2.76	-	-	-	-	-	-
$HG_{Ser105} – O3_{subs}$	2.96	2.97	3.22	3.31	2.42	0.98	-	-	-	-	-	-
$O_{wat} – C1_{subs}$	-	-	-	-	-	-	2.70	1.65	1.54	1.47	1.42	1.32
$O_{wat} – H1_{wat}$	-	-	-	-	-	-	0.98	1.37	1.61	2.77	2.68	3.86
$H1_{wat} – NE2_{His224}$	-	-	-	-	-	-	1.98	1.18	1.08	1.03	1.30	4.68
$H1_{wat} – OG_{Ser105}$	-	-	-	-	-	-	3.17	3.00	3.07	2.16	1.35	1.01
$HD_{His224} – OD2_{Asp187}$	1.70	1.74	1.59	1.55	1.65	1.82	1.89	1.81	1.76	1.58	1.70	1.93
$HD_{His224} – ND_{His224}$	1.04	1.05	1.07	1.09	1.07	1.03	1.03	1.04	1.05	1.08	1.05	1.03
$C1_{subs} – O2_{subs}$	1.23	1.27	1.30	1.30	1.29	1.23	1.23	1.28	1.30	1.30	1.27	1.23
$O2_{subs} – H_{Thr40}$	1.93	2.00	1.90	1.93	1.66	1.78	1.63	1.66	1.64	1.72	1.86	1.67
$O2_{subs} – HG1_{Thr40}$	1.56	1.61	1.53	1.57	1.66	1.64	1.81	1.78	1.73	1.57	1.66	1.71
$O2_{subs} – H_{Gln106}$	1.92	1.85	1.77	1.85	2.28	2.29	2.21	2.68	2.65	1.98	2.00	2.60

hydrolysis of MHET at pH 5

	RC	TS1	INT1	INT1	TS2	INT2	INT2	TS3	INT3	INT3	TS4	PC
$OG_{Ser105} – C1_{subs}$	2.26	1.74	1.50	1.45	1.41	1.33	1.32	1.41	1.45	1.48	1.69	2.42
$OG_{Ser105} – HG_{Ser105}$	1.00	1.29	1.87	2.52	2.51	2.58	-	-	-	-	-	-
$NE2_{His224} – HG_{Ser105}$	1.71	1.28	1.04	1.07	1.18	1.78	-	-	-	-	-	-
$C1_{subs} – O3_{subs}$	1.33	1.40	1.45	1.55	1.74	2.65	-	-	-	-	-	-
$HG_{Ser105} – O3_{subs}$	2.99	3.00	3.20	1.60	1.35	0.99	-	-	-	-	-	-
$O_{wat} – C1_{subs}$	-	-	-	-	-	-	2.55	1.64	1.46	1.45	1.41	1.34
$O_{wat} – H1_{wat}$	-	-	-	-	-	-	0.99	1.17	1.81	3.08	2.77	2.78
$H1_{wat} – NE2_{His224}$	-	-	-	-	-	-	1.85	1.37	1.05	1.03	1.32	2.02
$H1_{wat} – OG_{Ser105}$	-	-	-	-	-	-	2.80	2.59	2.65	1.96	1.25	0.97
$HD_{His224} – OD2_{Asp87}$	1.63	1.60	1.49	1.64	1.74	1.76	1.81	1.82	1.61	1.42	1.63	1.66
$HD_{His224} – ND_{His224}$	1.05	1.06	1.09	1.07	1.05	1.04	1.03	1.04	1.07	1.11	1.06	1.05
$C1_{subs} – O2_{subs}$	1.24	1.27	1.30	1.29	1.27	1.23	1.23	1.28	1.31	1.30	1.27	1.23
$O2_{subs} – H_{Thr40}$	1.76	1.84	1.77	1.58	1.60	1.80	1.77	1.62	1.61	1.69	1.74	1.65
$O2_{subs} – HG1_{Thr40}$	1.61	1.70	1.66	1.94	1.94	1.78	1.75	1.79	1.71	1.66	1.72	1.74
$O2_{subs} – H_{Gln106}$	1.83	1.76	1.72	2.35	2.32	1.92	1.90	2.22	2.14	1.77	1.82	2.04

Supplementary Table 6: ESP atomic charges (in a.u.) computed on the M06-2X/MM optimized geometries along the reaction profile using the CHelpG method.

hydrolysis of BHET at pH 5						
	$C1_{subs}$	$O3_{subs}$	$O2_{subs}$	$NE2_{His224}$	OG_{Ser105}	O_{wat}
RC	0.543	-0.383	-0.731	-0.284	-0.594	n.a
TS1	0.683	-0.472	-0.815	-0.010	-0.822	n.a
INT1	0.861/0.646	-0.615/-0.510	-1.031/-0.943	0.042/0.092	-0.711/-0.485	n.a
TS2	0.598	-0.532	-0.879	0.068	-0.442	n.a
INT2	0.688/0.729	-0.617/n.a	-0.754/-0.774	-0.231/-0.319	-0.394/-0.417	n.a/-0.707
TS3	0.672	n.a	-0.903	-0.112	-0.486	-0.736
INT3	0.857/0.968	n.a	-1.045/-1.018	-0.084/-0.024	-0.631/-0.799	-0.830/-0.704
TS4	0.957	n.a	-0.908	0.037	-0.680	-0.687
PC	0.689	n.a	-0.735	-0.244	-0.538	-0.538

hydrolysis of BHET at pH 9						
	$C1_{subs}$	$O3_{subs}$	$O2_{subs}$	$NE2_{His224}$	OG_{Ser105}	O_{wat}
RC	0.478	-0.414	-0.736	-0.257	-0.489	n.a
TS1	0.728	-0.587	-0.940	0.062	-0.483	n.a
INT1	0.838/0.838	-0.635/-0.650	-1.035/-1.021	0.177/0.072	-0.594/-0.544	n.a
TS2	1.005	-0.603	-1.075	0.053	-0.636	n.a
INT2	0.755/0.809	-0.445/n.a	-0.810/-0.838	-0.259/-0.273	-0.356/-0.341	n.a/-0.815
TS3	0.763	n.a	-0.985	-0.001	-0.480	-0.726
INT3	0.787/0.959	n.a	-1.038/-1.057	0.007/0.053	-0.510/-0.688	-0.783/-0.738
TS4	0.867	n.a	-0.964	0.073	-0.554	-0.725
PC	0.808	n.a	-0.826	-0.283	-0.727	-0.509

hydrolysis of $MHET^{(-)}$ at pH 5						
	$C1_{subs}$	$O3_{subs}$	$O2_{subs}$	$NE2_{His224}$	OG_{Ser105}	O_{wat}
RC	0.430	-0.371	-0.755	-0.145	-0.451	n.a
TS1	0.587	-0.522	-0.898	0.031	-0.430	n.a
INT1	0.691/0.788	-0.589/-0.528	-1.005/-1.006	0.187/0.090	-0.513/-0.534	n.a
TS2	0.756	-0.503	-0.940	0.061	-0.491	n.a
INT2	0.843/0.830	-0.327/n.a	-0.828/-0.842	-0.157/-0.213	-0.438/-0.403	n.a/-0.860
TS3	0.858	n.a	-1.014	-0.128	-0.517	-0.752
INT3	0.901/0.928	n.a	-1.108/-1.043	-0.009/0.152	-0.632/-0.597	-0.737/-0.843
TS4	0.786	n.a	-0.934	0.003	-0.455	-0.751
PC	0.615	n.a	-0.757	-0.262	-0.488	-0.548

Supplementary Table 7. Cartesian coordinates (in Å) of QM atoms and imaginary frequency (in cm^{-1}) for Transition State Structures for the hydrolysis of **BHET** catalysed by CALB at **pH 5**, optimized at M06-2X/MM level.

Transition state 1 ($v^{\ddagger}$ = 661.681i)					Transition state 2 ($v^{\ddagger}$ = 297.576i)				
	**Atom**	**x**	**y**	**z**		**Atom**	**x**	**y**	**z**
**1**	C	47.349951	42.085696	45.607653	**1**	C	47.220548	42.111657	45.449273
**2**	H	48.225514	42.552510	46.073825	**2**	H	47.919830	42.443563	46.224219
**3**	H	46.917080	42.890002	44.988224	**3**	H	46.970751	42.998256	44.856722
**4**	O	46.448390	41.802008	46.685584	**4**	O	46.047149	41.594350	46.073049
**5**	H	47.145250	41.982482	48.102583	**5**	H	46.979411	42.601886	48.523818
**6**	C	50.307332	37.836605	54.013031	**6**	C	50.295083	37.840073	53.981728
**7**	H	51.378415	37.722644	53.839332	**7**	H	51.367176	37.716198	53.811441
**8**	H	49.842040	36.843836	54.013429	**8**	H	49.827360	36.847819	53.966327
**9**	C	49.676634	38.633334	52.855679	**9**	C	49.684837	38.634472	52.809496
**10**	O	48.476482	38.955386	52.879133	**10**	O	48.481033	38.953431	52.827418
**11**	O	50.446677	38.928392	51.874548	**11**	O	50.462600	38.925171	51.835502
**12**	C	49.183766	42.185582	52.623406	**12**	C	49.237068	42.120033	52.707349
**13**	H	48.783838	41.343361	53.198831	**13**	H	48.901635	41.228192	53.245959
**14**	H	48.682566	43.082386	53.000606	**14**	H	48.729579	42.976753	53.157986
**15**	C	48.727722	41.960694	51.157208	**15**	C	48.756937	41.995140	51.275965
**16**	N	49.025324	40.783679	50.498945	**16**	N	48.966649	40.884718	50.483547
**17**	H	49.580239	40.002642	50.933128	**17**	H	49.498110	40.050890	50.810421
**18**	C	48.436008	40.767821	49.299602	**18**	C	48.326692	41.041935	49.316569
**19**	H	48.505993	39.967724	48.576450	**19**	H	48.315157	40.323580	48.508291
**20**	N	47.747825	41.887956	49.104018	**20**	N	47.711027	42.220995	49.305382
**21**	C	47.925157	42.653392	50.263341	**21**	C	47.980759	42.830232	50.515408
**22**	H	47.447807	43.614541	50.378093	**22**	H	47.587230	43.800545	50.755258
**23**	O	43.594171	45.876867	48.516979	**23**	O	43.682960	45.977920	48.489586
**24**	H	43.218943	46.285188	47.713843	**24**	H	43.178775	46.502289	47.842551
**25**	C	45.005187	45.754105	48.396360	**25**	C	44.844818	45.481632	47.809477
**26**	H	45.439886	46.010051	49.370564	**26**	H	45.616478	46.259291	47.770875
**27**	H	45.393482	46.460742	47.656506	**27**	H	44.585599	45.199865	46.788961
**28**	C	45.460240	44.348402	48.030826	**28**	C	45.364522	44.240709	48.546842
**29**	H	46.557951	44.310325	48.055815	**29**	H	46.144588	44.551220	49.257693
**30**	H	45.057923	43.609001	48.732851	**30**	H	44.543715	43.822257	49.146172
**31**	O	45.040560	44.089899	46.703488	**31**	O	45.946333	43.259375	47.705880
**32**	C	44.701763	42.845667	46.255664	**32**	C	45.076616	42.548162	46.398643
**33**	O	44.568748	42.785484	45.020509	**33**	O	44.776379	43.403814	45.513686
**34**	C	43.781855	42.010800	47.123472	**34**	C	43.919502	41.833476	47.080990
**35**	C	42.855998	42.638767	47.965651	**35**	C	42.919910	42.561013	47.739939
**36**	C	41.873363	41.893413	48.614815	**36**	C	41.869678	41.904885	48.374162
**37**	H	41.161368	42.381805	49.272238	**37**	H	41.130936	42.467620	48.936334
**38**	H	42.888912	43.714428	48.114817	**38**	H	42.961147	43.645981	47.772935
**39**	C	43.698640	40.631211	46.916342	**39**	C	43.784369	40.448786	46.961988
**40**	H	44.418274	40.166285	46.252306	**40**	H	44.530701	39.894329	46.406977
**41**	C	42.704604	39.888562	47.544599	**41**	C	42.697972	39.794862	47.531940
**42**	H	42.618525	38.819709	47.371485	**42**	H	42.573180	38.722682	47.414724
**43**	C	41.793082	40.515508	48.403260	**43**	C	41.763515	40.513043	48.277640
**44**	C	40.754111	39.686711	49.080259	**44**	C	40.702562	39.741659	48.976911
**45**	O	40.471195	38.546267	48.782991	**45**	O	40.368763	38.610981	48.692985
**46**	O	40.159762	40.346777	50.094028	**46**	O	40.175831	40.422533	50.013197
**47**	C	39.069486	39.699186	50.767344	**47**	C	39.145540	39.765533	50.766723
**48**	H	38.559781	40.506440	51.295417	**48**	H	38.687168	40.562176	51.354607
**49**	H	38.391704	39.249996	50.035655	**49**	H	38.406852	39.330130	50.088499
**50**	C	39.566370	38.642226	51.741087	**50**	C	39.731789	38.695093	51.671909
**51**	H	38.737926	38.338242	52.386850	**51**	H	38.948236	38.321655	52.336256
**52**	H	39.932291	37.764745	51.199579	**52**	H	40.118114	37.862490	51.076327
**53**	O	40.588521	39.146824	52.598684	**53**	O	40.760748	39.217580	52.511286
**54**	H	41.406155	39.202423	52.090491	**54**	H	41.552559	39.343681	51.975323
**55**	H	50.161690	42.246544	52.823275	**55**	H	50.221860	42.213717	52.853666
**56**	H	50.200371	38.239431	54.922036	**56**	H	50.181708	38.238955	54.891694
**57**	H	47.707121	41.395650	44.978156	**57**	H	47.657763	41.417084	44.877948

Transition state 3 ($\nu^‡$ = 777.043i)					Transition state 4 ($\nu^‡$ = 1240.238i)				
	Atom	x	y	z		Atom	x	y	z
**1**	C	47.153964	42.041455	45.380072	**1**	C	47.334794	42.285743	45.621645
**2**	H	47.825885	42.344689	46.190704	**2**	H	48.192055	42.744516	46.121038
**3**	H	46.902039	42.936854	44.802557	**3**	H	46.875588	43.077091	45.022028
**4**	O	45.974177	41.469634	45.940862	**4**	O	46.416699	41.911928	46.672566
**5**	H	46.796672	42.649979	48.501259	**5**	H	47.049573	42.003181	47.892331
**6**	C	50.382535	37.912384	53.968168	**6**	C	50.286941	37.877167	53.992816
**7**	H	51.456613	37.861431	53.780650	**7**	H	51.358879	37.762413	53.823391
**8**	H	49.978992	36.893044	53.936528	**8**	H	49.820489	36.885242	53.975788
**9**	C	49.691518	38.682183	52.810872	**9**	C	49.667353	38.682658	52.834177
**10**	O	48.486539	38.979817	52.868587	**10**	O	48.469660	39.018440	52.860928
**11**	O	50.408006	38.955753	51.786736	**11**	O	50.438640	38.956867	51.849566
**12**	C	49.206883	42.219357	52.743494	**12**	C	49.177334	42.243070	52.505659
**13**	H	48.853360	41.347368	53.302594	**13**	H	48.750132	41.416123	53.082722
**14**	H	48.723696	43.097368	53.182372	**14**	H	48.685405	43.154822	52.858963
**15**	C	48.702401	42.085293	51.314678	**15**	C	48.724180	42.007973	51.035930
**16**	N	48.935215	40.976491	50.527487	**16**	N	48.983376	40.810235	50.399420
**17**	H	49.476106	40.148914	50.847009	**17**	H	49.524881	40.032519	50.839464
**18**	C	48.275064	41.110315	49.366529	**18**	C	48.378047	40.787352	49.204563
**19**	H	48.281678	40.380660	48.568356	**19**	H	48.431871	39.964877	48.505335
**20**	N	47.619642	42.264846	49.347202	**20**	N	47.713472	41.918101	48.986046
**21**	C	47.888935	42.887857	50.549957	**21**	C	47.930123	42.698471	50.129834
**22**	H	47.456568	43.845598	50.779874	**22**	H	47.490505	43.680105	50.224940
**23**	H	46.235973	43.938348	47.289885	**23**	H	45.574240	43.740542	47.971285
**24**	O	45.899293	43.130773	47.705129	**24**	O	45.231292	43.981235	47.100845
**25**	C	45.039288	42.404306	46.363630	**25**	C	44.900148	42.800460	46.417775
**26**	O	44.749911	43.354438	45.579552	**26**	O	44.700052	42.970107	45.185093
**27**	C	43.899449	41.691704	47.068769	**27**	C	43.892870	41.932032	47.161745
**28**	C	43.050541	42.407493	47.918532	**28**	C	42.999209	42.541235	48.048720
**29**	C	41.975886	41.775769	48.534136	**29**	C	41.970546	41.804328	48.631674
**30**	H	41.334603	42.323898	49.216367	**30**	H	41.278920	42.279763	49.318637
**31**	H	43.251406	43.454553	48.120875	**31**	H	43.090611	43.599618	48.278672
**32**	C	43.627302	40.351576	46.790688	**32**	C	43.726239	40.580550	46.838530
**33**	H	44.267688	39.807268	46.106261	**33**	H	44.402338	40.121239	46.124791
**34**	C	42.540486	39.719445	47.388976	**34**	C	42.699530	39.841442	47.418511
**35**	H	42.321167	38.676436	47.181161	**35**	H	42.561128	38.791600	47.174563
**36**	C	41.729099	40.420013	48.280839	**36**	C	41.824363	40.449457	48.327467
**37**	C	40.653290	39.663577	48.979402	**37**	C	40.765465	39.625726	48.981531
**38**	O	40.293572	38.543462	48.686718	**38**	O	40.462611	38.496185	48.665929
**39**	O	40.140089	40.348943	50.019340	**39**	O	40.187207	40.283220	50.006583
**40**	C	39.094495	39.714875	50.771675	**40**	C	39.113631	39.638789	50.707810
**41**	H	38.624303	40.528059	51.326580	**41**	H	38.582248	40.453884	51.201363
**42**	H	38.368893	39.257438	50.093899	**42**	H	38.449517	39.135267	49.999610
**43**	C	39.670402	38.673428	51.716294	**43**	C	39.654194	38.646106	51.724465
**44**	H	38.884775	38.330500	52.394680	**44**	H	38.844050	38.341855	52.392678
**45**	H	40.050794	37.815849	51.152402	**45**	H	40.047423	37.757900	51.218999
**46**	O	40.704147	39.216720	52.535037	**46**	O	40.665727	39.223099	52.546526
**47**	H	41.486567	39.347571	51.986126	**47**	H	41.459016	39.333221	52.009220
**48**	H	50.194348	42.292496	52.883364	**48**	H	50.151294	42.287372	52.728012
**49**	H	50.263376	38.266519	54.895740	**49**	H	50.177752	38.263858	54.908539
**50**	H	47.627266	41.380951	44.797217	**50**	H	47.678686	41.574673	45.008360

Supplementary Table 8. Cartesian coordinates (in Å) of QM atoms and imaginary frequency (in cm^{-1}) for Transition State Structures for the hydrolysis of **BHET** catalysed by CALB at **pH 9**, optimized at M06-2X/MM level.

Transition state 1 ($\nu^{‡}$ = 1214.242i)					Transition state 2 ($\nu^{‡}$ = 135.889i)				
	**Atom**	**x**	**y**	**z**		**Atom**	**x**	**y**	**z**
**1**	C	52.514488	45.258857	43.534152	**1**	C	52.222069	45.172088	43.616679
**2**	H	53.509230	45.201419	43.081111	**2**	H	53.283068	45.220160	43.328453
**3**	H	51.904755	45.866048	42.862888	**3**	H	51.678063	45.718761	42.839185
**4**	O	52.728203	46.031822	44.753150	**4**	O	52.068902	45.842699	44.865962
**5**	H	54.050610	46.116957	45.097828	**5**	H	54.709142	46.532328	45.063460
**6**	C	59.756868	43.168511	50.055409	**6**	C	59.717438	43.168911	50.038012
**7**	H	60.178809	42.318588	49.515096	**7**	H	60.138479	42.330802	49.477197
**8**	H	59.211265	42.772451	50.919483	**8**	H	59.184774	42.745845	50.897551
**9**	C	58.709058	43.840927	49.140403	**9**	C	58.651270	43.835049	49.149586
**10**	O	58.276207	44.987044	49.420837	**10**	O	58.243762	44.992854	49.417305
**11**	O	58.311295	43.180055	48.137538	**11**	O	58.206361	43.162105	48.168020
**12**	C	58.863011	46.384652	46.691209	**12**	C	59.066546	46.419188	46.785812
**13**	H	58.856923	46.262207	47.777996	**13**	H	59.085071	46.279526	47.869305
**14**	H	59.052238	47.442656	46.483860	**14**	H	59.309956	47.465250	46.580036
**15**	C	57.425683	46.054938	46.212962	**15**	C	57.648914	46.156627	46.324512
**16**	N	56.875494	44.804026	46.421546	**16**	N	56.991763	44.958962	46.554314
**17**	H	57.342150	44.072165	47.005619	**17**	H	57.390155	44.173339	47.159023
**18**	C	55.613163	44.788402	45.964373	**18**	C	55.763438	45.021465	46.041383
**19**	H	54.953124	43.933046	45.996434	**19**	H	55.006138	44.250069	46.063925
**20**	N	55.268946	45.972471	45.472251	**20**	N	55.592367	46.221670	45.491161
**21**	C	56.404151	46.778857	45.618657	**21**	C	56.755991	46.950951	45.654274
**22**	H	56.397869	47.813089	45.307843	**22**	H	56.844722	47.966191	45.302669
**23**	O	53.222955	51.076308	44.144323	**23**	O	52.935119	51.078949	44.249975
**24**	H	52.375440	51.222272	43.679425	**24**	H	52.168601	51.274420	43.671863
**25**	C	53.965524	50.047567	43.525851	**25**	C	53.797078	50.121641	43.670694
**26**	H	55.027285	50.307686	43.633511	**26**	H	54.810398	50.554819	43.687027
**27**	H	53.740204	49.986392	42.454313	**27**	H	53.532481	49.913776	42.628048
**28**	C	53.749591	48.672271	44.148305	**28**	C	53.877163	48.779707	44.414851
**29**	H	54.436511	47.957294	43.666864	**29**	H	54.872917	48.388988	44.124731
**30**	H	53.972689	48.704605	45.223849	**30**	H	53.896997	48.960936	45.500342
**31**	O	52.414774	48.291065	43.905423	**31**	O	52.940371	47.815043	44.016452
**32**	C	51.738432	47.365017	44.743040	**32**	C	51.700013	47.230251	44.720969
**33**	O	50.635393	47.033786	44.201314	**33**	O	50.680689	47.388515	43.939986
**34**	C	51.661588	47.839644	46.211539	**34**	C	51.544457	47.788663	46.154982
**35**	C	51.882570	49.193404	46.522107	**35**	C	51.672955	49.163182	46.418017
**36**	C	51.691084	49.681063	47.812369	**36**	C	51.529873	49.677206	47.703912
**37**	H	51.898816	50.723375	48.033828	**37**	H	51.690835	50.736124	47.884479
**38**	H	52.208267	49.888518	45.755975	**38**	H	51.906497	49.855136	45.618084
**39**	C	51.175282	46.993970	47.216754	**39**	C	51.148205	46.954234	47.208212
**40**	H	50.877545	45.958382	47.019425	**40**	H	50.908780	45.901589	47.050604
**41**	C	50.960885	47.491935	48.505318	**41**	C	50.948921	47.476394	48.488807
**42**	H	50.573537	46.827777	49.270522	**42**	H	50.621287	46.813317	49.282339
**43**	C	51.240563	48.827185	48.823724	**43**	C	51.183681	48.828378	48.762021
**44**	C	51.106884	49.282204	50.237261	**44**	C	51.106795	49.295100	50.172138
**45**	O	50.532871	48.675021	51.114997	**45**	O	50.572122	48.695010	51.080103
**46**	O	51.726256	50.470023	50.479913	**46**	O	51.740001	50.483866	50.385268
**47**	C	51.608259	50.993334	51.807473	**47**	C	51.675307	50.997138	51.719432
**48**	H	51.609152	52.082685	51.691615	**48**	H	51.710847	52.087183	51.615747
**49**	H	50.656633	50.677161	52.241918	**49**	H	50.724545	50.708407	52.174142
**50**	C	52.740212	50.575411	52.736288	**50**	C	52.814901	50.531575	52.617073
**51**	H	52.441541	50.855774	53.757907	**51**	H	52.526769	50.756971	53.654777
**52**	H	52.869372	49.490531	52.700783	**52**	H	52.943197	49.449891	52.523476
**53**	O	53.999101	51.153114	52.419513	**53**	O	54.072398	51.129155	52.322975
**54**	H	53.870459	52.084931	52.135403	**54**	H	53.933889	52.063479	52.063529
**55**	H	59.614316	45.829819	46.333856	**55**	H	59.762049	45.820661	46.388268
**56**	H	60.522198	43.726982	50.375376	**56**	H	60.479032	43.730792	50.360911
**57**	H	52.137811	44.333053	43.565797	**57**	H	51.940429	44.212568	43.615446

Transition state 3 ($\nu^{\ddagger}$ = 482.209i)					Transition state 4 ($\nu^{\ddagger}$ = 1241.800i)				
	Atom	x	y	z		Atom	x	y	z
1	C	52.160415	45.088722	43.651764	1	C	52.540276	45.216408	43.539535
2	H	53.239845	45.170291	43.478311	2	H	53.546676	45.153560	43.112930
3	H	51.671987	45.661695	42.856248	3	H	51.955233	45.847547	42.870481
4	O	51.848577	45.654763	44.923203	4	O	52.707585	45.922307	44.798513
5	H	54.558921	46.937712	45.091778	5	H	54.021761	46.027030	45.110074
6	C	59.749001	43.165123	49.992922	6	C	59.772735	43.165027	50.012943
7	H	60.185190	42.349804	49.409743	7	H	60.212496	42.337948	49.451738
8	H	59.206289	42.712215	50.830334	8	H	59.224030	42.737019	50.859319
9	C	58.685694	43.852252	49.103503	9	C	58.725445	43.845476	49.104694
10	O	58.297459	45.018769	49.375135	10	O	58.307088	44.998955	49.375275
11	O	58.225312	43.187538	48.131722	11	O	58.310041	43.173902	48.114148
12	C	59.104908	46.463969	46.743402	12	C	58.872296	46.390992	46.639684
13	H	59.104250	46.330102	47.827360	13	H	58.866827	46.303442	47.729372
14	H	59.390295	47.500725	46.537653	14	H	59.055610	47.443595	46.399452
15	C	57.672960	46.279112	46.271013	15	C	57.432345	46.042164	46.174163
16	N	56.944102	45.130001	46.508967	16	N	56.899044	44.789355	46.411292
17	H	57.299570	44.324120	47.066287	17	H	57.380383	44.068382	47.003847
18	C	55.699633	45.293113	46.032488	18	C	55.632035	44.752102	45.972961
19	H	54.903773	44.563122	46.087548	19	H	54.987296	43.886557	46.023098
20	N	55.578606	46.498411	45.492862	20	N	55.264355	45.922703	45.465201
21	C	56.802043	47.126342	45.627079	21	C	56.393581	46.744309	45.579936
22	H	56.956372	48.135218	45.276605	22	H	56.374121	47.770558	45.243721
23	H	53.360944	48.335907	44.313170	23	H	53.602002	48.246196	44.537215
24	O	53.370599	47.413632	44.607695	24	O	52.800503	48.137202	44.007237
25	C	51.787133	47.048693	44.897915	25	C	51.872497	47.329918	44.723187
26	O	51.085569	47.574464	43.967235	26	O	50.796971	47.156764	44.060262
27	C	51.565795	47.603075	46.307196	27	C	51.718405	47.794343	46.181791
28	C	51.812354	48.970568	46.519037	28	C	51.999842	49.133971	46.501481
29	C	51.620831	49.543792	47.768696	29	C	51.762315	49.631263	47.781459
30	H	51.847378	50.592458	47.931600	30	H	52.000077	50.663372	48.017524
31	H	52.172894	49.589406	45.698916	31	H	52.398697	49.805828	45.745534
32	C	51.062273	46.830694	47.352292	32	C	51.153158	46.970568	47.162678
33	H	50.736599	45.792008	47.214792	33	H	50.824952	45.945417	46.962647
34	C	50.847028	47.414037	48.604548	34	C	50.913421	47.472002	48.444099
35	H	50.448877	46.806090	49.410062	35	H	50.477589	46.816795	49.191597
36	C	51.152199	48.756439	48.832061	36	C	51.231605	48.796244	48.770103
37	C	51.044353	49.271466	50.220751	37	C	51.058818	49.251891	50.180592
38	O	50.491052	48.699301	51.135618	38	O	50.450775	48.646410	51.034979
39	O	51.672932	50.464066	50.404400	39	O	51.684751	50.430398	50.442591
40	C	51.628856	50.991854	51.732538	40	C	51.552897	50.946233	51.772420
41	H	51.663898	52.080075	51.615824	41	H	51.519763	52.035327	51.661487
42	H	50.685916	50.707853	52.205931	42	H	50.612183	50.598465	52.206697
43	C	52.785803	50.526057	52.605967	43	C	52.698945	50.552032	52.693979
44	H	52.529084	50.769113	53.647983	44	H	52.401590	50.831714	53.716293
45	H	52.897385	49.441044	52.523051	45	H	52.842256	49.468402	52.662999
46	O	54.040842	51.101694	52.266022	46	O	53.948364	51.142341	52.367436
47	H	53.907251	52.036933	51.998265	47	H	53.813039	52.072871	52.079365
48	H	59.790228	45.842579	46.363652	48	H	59.625695	45.828074	46.299811
49	H	60.503570	43.717820	50.346688	49	H	60.527860	43.721398	50.359696
50	H	51.896175	44.125650	43.600097	50	H	52.145173	44.297882	43.553815

Supplementary Table 9. Cartesian coordinates (in Å) of QM atoms and imaginary frequency (in cm^{-1}) for Transition State Structures for the hydrolysis of **MHET$^{(-)}$** catalysed by CALB at **pH 5**, optimized at M06-2X/MM level.

**Transition state 1 (ν‡ = -1050.101 cm^{-1})**					**Transition state 2 (ν‡ = -411.050 cm^{-1})**				
**Lp**	**Atom**	**x**	**y**	**z**	**Lp**	**Atom**	**x**	**y**	**z**
**1**	C	48.886520	43.216823	45.670153	**1**	C	48.755701	42.995354	45.683828
**2**	H	49.884650	43.600008	45.928278	**2**	H	49.666811	43.291154	46.213053
**3**	H	48.447495	43.927719	44.964132	**3**	H	48.516417	43.801209	44.986336
**4**	O	48.108493	43.260660	46.885950	**4**	O	47.690967	42.867160	46.649513
**5**	H	48.915454	43.361720	47.883307	**5**	H	49.143665	44.174719	48.232410
**6**	C	52.752128	39.537519	53.746367	**6**	C	52.713855	39.509799	53.726105
**7**	H	53.735671	39.521053	53.267053	**7**	H	53.697539	39.479004	53.246636
**8**	H	52.427643	38.499990	53.892802	**8**	H	52.375806	38.477051	53.875489
**9**	C	51.769644	40.197800	52.772622	**9**	C	51.737208	40.180126	52.745957
**10**	O	50.855551	40.932400	53.199582	**10**	O	50.826515	40.914855	53.188233
**11**	O	51.954657	39.951424	51.535332	**11**	O	51.915219	39.949783	51.507900
**12**	C	52.123788	43.747620	51.748359	**12**	C	52.193975	43.711789	51.908180
**13**	H	51.838358	43.082116	52.570877	**13**	H	51.946414	42.971270	52.676237
**14**	H	51.839059	44.754573	52.066041	**14**	H	51.941265	44.685766	52.335013
**15**	C	51.253261	43.366511	50.543326	**15**	C	51.299481	43.475127	50.719066
**16**	N	51.026839	42.051292	50.186255	**16**	N	50.958113	42.227619	50.236613
**17**	H	51.383906	41.191841	50.698541	**17**	H	51.258422	41.315952	50.660818
**18**	C	50.161799	42.019069	49.165326	**18**	C	50.101945	42.379549	49.214709
**19**	H	49.815953	41.111841	48.690292	**19**	H	49.652286	41.572399	48.655042
**20**	N	49.788752	43.245200	48.815431	**20**	N	49.876363	43.670091	49.004675
**21**	C	50.470354	44.099835	49.672716	**21**	C	50.616119	44.365818	49.933319
**22**	H	50.347144	45.170583	49.613015	**22**	H	50.596380	45.443113	49.980781
**23**	O	47.296699	48.186575	47.790352	**23**	O	47.338874	48.351500	47.544036
**24**	H	46.622552	48.306970	47.097139	**24**	H	46.550041	48.666845	47.064836
**25**	C	48.465969	47.576810	47.286155	**25**	C	47.897153	47.265527	46.816712
**26**	H	49.309743	47.955501	47.874095	**26**	H	48.654167	47.628592	46.109062
**27**	H	48.642512	47.853011	46.239244	**27**	H	47.121795	46.738964	46.255314
**28**	C	48.457349	46.059093	47.390714	**28**	C	48.526610	46.277151	47.802045
**29**	H	49.439139	45.665192	47.088025	**29**	H	49.608281	46.462394	47.875086
**30**	H	48.253617	45.737705	48.418995	**30**	H	48.088034	46.456060	48.793953
**31**	O	47.463501	45.591866	46.496759	**31**	O	48.382152	44.923597	47.409229
**32**	C	46.794106	44.383881	46.695674	**32**	C	46.988757	44.073095	46.819066
**33**	O	46.165510	44.042636	45.645504	**33**	O	46.524315	44.575780	45.752228
**34**	C	46.014578	44.302448	48.013086	**34**	C	46.056841	44.073413	48.025538
**35**	C	45.315279	43.131333	48.327356	**35**	C	45.409308	42.912380	48.448541
**36**	H	45.417350	42.265124	47.681227	**36**	H	45.614597	41.974801	47.943512
**37**	C	44.462596	43.090344	49.428789	**37**	C	44.507970	42.954319	49.514540
**38**	H	43.943598	42.175221	49.694129	**38**	H	44.032582	42.045980	49.868210
**39**	C	44.271563	44.219959	50.235228	**39**	C	44.251244	44.145908	50.199953
**40**	C	43.482835	44.119068	51.529625	**40**	C	43.476093	44.115981	51.508431
**41**	O	43.215631	45.182141	52.161394	**41**	O	43.242184	45.205096	52.106682
**42**	O	43.197790	42.956604	51.919691	**42**	O	43.170432	42.975174	51.947818
**43**	C	44.926814	45.399393	49.892366	**43**	C	44.848165	45.318871	49.733609
**44**	H	44.771481	46.286572	50.498972	**44**	H	44.645686	46.256380	50.243553
**45**	C	45.788042	45.443124	48.794714	**45**	C	45.722974	45.284828	48.649862
**46**	H	46.255405	46.389099	48.547855	**46**	H	46.142109	46.221756	48.300762
**47**	H	53.119523	43.710854	51.663735	**47**	H	53.181649	43.681522	51.754613
**48**	H	52.846610	39.980235	54.638037	**48**	H	52.814309	39.956733	54.615014
**49**	H	49.016375	42.340963	45.205387	**49**	H	48.957087	42.160506	45.171508

Transition state 3 (ν‡ = -603.182 cm^{-1})					Transition state 4 (ν‡ = -1103.139 cm^{-1})				
**Lp**	**Atom**	**x**	**y**	**z**	**Lp**	**Atom**	**x**	**y**	**z**
**1**	C	48.789805	42.889998	45.679152	**1**	C	48.884447	43.178913	45.669298
**2**	H	49.699605	43.136073	46.237697	**2**	H	49.875381	43.550930	45.958354
**3**	H	48.575907	43.728859	45.014206	**3**	H	48.458869	43.909302	44.979677
**4**	O	47.709655	42.747475	46.622916	**4**	O	48.080228	43.174018	46.873408
**5**	H	48.962805	44.447934	48.116172	**5**	H	48.867569	43.283799	47.842196
**6**	C	52.786831	39.585407	53.693152	**6**	C	52.737167	39.533177	53.753669
**7**	H	53.769682	39.580688	53.211245	**7**	H	53.722502	39.526614	53.275899
**8**	H	52.465307	38.544229	53.823640	**8**	H	52.419599	38.493799	53.894578
**9**	C	51.790213	40.256991	52.726292	**9**	C	51.759253	40.198500	52.777390
**10**	O	50.848345	40.940462	53.189443	**10**	O	50.874456	40.970387	53.200925
**11**	O	51.982375	40.071024	51.485974	**11**	O	51.920120	39.919906	51.543816
**12**	C	52.221673	43.779696	51.891958	**12**	C	52.132389	43.795586	51.710730
**13**	H	51.938117	43.036152	52.645134	**13**	H	51.823191	43.170099	52.555979
**14**	H	52.005067	44.753211	52.340416	**14**	H	51.864631	44.820922	51.982635
**15**	C	51.300714	43.606749	50.700314	**15**	C	51.261167	43.385769	50.512886
**16**	N	50.898658	42.380230	50.213822	**16**	N	51.055425	42.062174	50.173217
**17**	H	51.193703	41.465147	50.610909	**17**	H	51.411063	41.212130	50.693735
**18**	C	50.009072	42.581233	49.221650	**18**	C	50.182291	42.001244	49.159919
**19**	H	49.515948	41.789349	48.675143	**19**	H	49.855780	41.079938	48.698391
**20**	N	49.809640	43.878288	49.024902	**20**	N	49.779717	43.214066	48.797437
**21**	C	50.612973	44.524938	49.941716	**21**	C	50.454359	44.092108	49.639802
**22**	H	50.634589	45.601767	50.007969	**22**	H	50.311010	45.159979	49.568012
**23**	H	48.060593	45.851815	47.278358	**23**	H	48.215818	45.585838	47.506033
**24**	O	48.276010	44.883792	47.268923	**24**	O	47.706618	45.516993	46.685498
**25**	C	47.014635	43.962927	46.776962	**25**	C	46.894545	44.366818	46.724003
**26**	O	46.512966	44.433590	45.696448	**26**	O	46.284554	44.173145	45.622362
**27**	C	46.077871	43.966142	47.987442	**27**	C	46.062011	44.265342	48.001962
**28**	C	45.299209	42.857642	48.323092	**28**	C	45.286735	43.125122	48.247899
**29**	H	45.397671	41.940929	47.750959	**29**	H	45.344844	42.283434	47.563841
**30**	C	44.404671	42.924501	49.393258	**30**	C	44.429016	43.080389	49.344672
**31**	H	43.838664	42.049885	49.697219	**31**	H	43.862047	42.182066	49.568460
**32**	C	44.252505	44.102066	50.131317	**32**	C	44.293639	44.185637	50.195929
**33**	C	43.468230	44.097305	51.425601	**33**	C	43.494674	44.082782	51.485688
**34**	O	43.228951	45.196840	52.003301	**34**	O	43.220878	45.148905	52.109199
**35**	O	43.167172	42.965128	51.881258	**35**	O	43.218680	42.919382	51.879879
**36**	C	44.981000	45.229695	49.754214	**36**	C	45.024186	45.338201	49.916754
**37**	H	44.865872	46.146364	50.324470	**37**	H	44.910428	46.200968	50.565013
**38**	C	45.887014	45.156668	48.701978	**38**	C	45.912117	45.376894	48.839109
**39**	H	46.467587	46.039939	48.450806	**39**	H	46.457352	46.298348	48.645518
**40**	H	53.210105	43.714048	51.755239	**40**	H	53.128023	43.733189	51.641310
**41**	H	52.883574	40.006686	54.594908	**41**	H	52.828376	39.974948	54.646148
**42**	H	48.982136	42.080360	45.124630	**42**	H	49.006420	42.309720	45.190105

Supplementary References

[1] Uppenberg, J., Morgens, H., Shamkant, P. & Alwyn, J. T. The Sequence, Crystal Structure Determination and Refinement of Two Crystal Forms of Lipase B from Candida Antarctica. *Structure* **2**, 293–308 (1994).

[2] Olsson, M. H. M., Søndergaard, C. R., Rostkowski, M. & Jensen, J. H. PROPKA3: Consistent Treatment of Internal and Surface Residues in Empirical pKa predictions, *J. Chem. Theory Comput.* **7**, 525–537 (2011).

[3] Mongan, J., Case, D. A. & McCammon, J. A. Constant pH Molecular Dynamics in Generalized Born Implicit Solvent, *J. Comput. Chem.* **25**, 2038–2048 (2004).

[4] Radak, B. K. et al. Constant-pH Molecular Dynamics Simulations for Large Biomolecular Systems, *J. Chem. Theory Comput.* **13**, 5933–5944 (2017).

[5] Phillips, J. C. et al. Scalable Molecular Dynamics with NAMD. *J. Comput. Chem.* **26**, 1781–1802 (2005).

[6] Huang, J. & MacKerell Jr, A. D. CHARMM36 all-atom additive protein force field: validation based on comparison to NMR data. *J Comput. Chem.* **30**, 2135-2145 (2013).

[7] Zhang, W. E. I. et al. A Point-Charge Force Field for Molecular Mechanics Simulations of Proteins Based on Condensed-Phase. *J. Comput. Chem.* **24**, 1999–2012 (2003).

[8] Phillips, J. C. et al. Scalable Molecular Dynamics with NAMD. *J. Comput. Chem.* **26**, 1781–1802 (2005).

[9] Wang, J., Wolf, R. M., Caldwell, J. W., Kollman, P. A. & Case, D. A. Development and Testing of a General Amber Force Field. J. Comput. Chem. **25**, 1157–1174 (2004).

[10] Wang, J., Wang, W., Kollman, P. A. & Case, D. A. Automatic Atom Type and Bond Type Perception in Molecular Mechanical Calculations. J. Mol. Graphics Modell. **25**, 247–260 (2006).

[11] Dewar, M. J. S., Zoebisch, E. G., Healy, E. F. & Stewart, J. J. P. Development and Use of Quantum Mechanical Molecular Models. 76. AM1: A New General Purpose Quantum Mechanical Molecular Model. *J. Am. Chem. Soc.* **107**, 3902–3909 (1985).

[12] Grest, G. S. & Kremer, K. Molecular Dynamics Simulation for Polymers in the Presence of a Heat Bath. *Phys. Rev. A*. **33**, 3628–3631 (1986).

[13] Roe, D. R. & Cheatham, III, T. E. PTRAJ and CPPTRAJ: Software for Processing and Analysis of Molecular Dynamics Trajectory Data. *J. Chem. Theory Comput.* **9**, 3084–3095 (2013).

[14] Case, D. A. et al. Amber 2021. University of California: San Francisco 2021.

[15] Zhao, Y. & Truhlar, D. G. The M06 Suite of Density Functionals for Main Group Thermochemistry, Thermochemical Kinetics, Noncovalent Interactions, Excited States, and Transition Elements: Two New Functionals and Systematic Testing of Four M06-Class Functionals and 12 Other Function, *Theor. Chem. Acc.* **120**, 215–241 (2008).

[16] Stewart, J. J. P. Quantum Chemistry Program Exchange 455. 1996, 6.

[17] Frisch, M. J. et al. Gaussian 09, Revision E.01. Gaussian, Inc.: Wallingford, CT 2009.

[18] Jorgensen, W. L., Maxwell, D. S. & Tirado-Rives, J. Development and Testing of the OPLS All-Atom Force Field on Conformational Energetics and Properties of Organic Liquids. *J. Am. Chem. Soc.* **118**, 11225–11236 (1996).

[19] Jorgensen, W. L., Chandrasekhar, J., Madura, J. D., Impey, R. W., & Klein, M. L. Comparison of Simple Potential Functions for Simulating Liquid Water, *J. Chem. Phys.* **79**, 926–935 (1983).

[20] Field, M. J., Albe, M., Bret, C., Proust-De Martin, F. & Thomas, A. The Dynamo Library for Molecular Simulations Using Hybrid Quantum Mechanical and Molecular Mechanical Potentials. *J. Comput. Chem.* **21**, 1088–1100 (2000).

[21] Byrd, R. H., Lu, P., Nocedal, J. & Zhu, C. A Limited Memory Algorithm for Bound Constrained Optimization. SIAM J. Sci. Comput. **16**, 1190–1208 (1995).

[22] Turner, A. J., Moliner, V. & Williams, I. H. Transition-State Structural Refinement with GRACE and CHARMM: Flexible QM/MM Modelling for Lactate Dehydrogenase. *Phys. Chem. Chem. Phys.* **1**, 1323–1331 (1999).
[23] Martí, S., Moliner, V. & Tuñón, I. Improving the QM/MM Description of Chemical Processes: A Dual Level Strategy to Explore the Potential Energy Surface in Very Large Systems. *J. Chem. Theory Comput.* **1**, 1008–1016 (2005).
[24] Baker, J., Kessi, A. & Delley, B. The Generation and Use of Delocalized Internal Coordinates in Geometry Optimization. *J. Chem. Phys.* **105**, 192–212 (1996).
[25] Baker, J. Constrained Optimization in Delocalized Internal Coordinates. *J. Comput. Chem.* **18**, 1079–1095 (1997).
[26] Roux, B. The Calculation of the Potential of Mean Force Using Computer-Simulation. *Comput. Phys. Commun.* **91**, 275–282 (1995)
[27] Roux, B. The Calculation of the Potential of Mean Force Using Computer-Simulation. *Comput. Phys. Commun.* **91**, 275–282 (1995).
[28] Torrie, G. M. & Valleau, J. P. Non-Physical Sampling Distibutions in Monte-Carlo Free-Energy Estimation - Umbrella Sampling. *J. Comput. Phys.* **23**, 187–199 (1977).
[29] Kumar, S., Rosenberg, J. M., Bouzida, D., Swendsen, R. H. & Kollman, P. A. The Weighted Histogram Analysis Method for Free-energy Calculations on Biomolecules. I. The Method. *J. Comput. Chem.* **13**, 1011–1021 (1992).
[30] Verlet, L. Computer "Experiments" on Classical Fluids. I. Thermodynamical Properties of Lennard-Jones Molecules. *Phys. Rev.* **159**, 98–103 (1967).
[31] Ruiz-Pernía, J. J., Silla, E., Tuñón, I., Martí, S. & Moliner, V. Hybrid QM/MM Potentials of Mean Force with Interpolated Corrections. *J. Phys. Chem. B* **108**, 8427–8433 (2004).
[32] Ruiz-Pernía, J. J., Silla, E., Tuñón, I. & Martí, S. Hybrid Quantum Mechanics/Molecular Mechanics Simulations with Two-Dimensional Interpolated Corrections: Application to Enzymatic Processes. *J. Phys. Chem. B* **110**, 17663–17670 (2006).
