# Supplementary material for: Mechanistic studies of a lipase unveil effect of pH on hydrolysis products of small PET modules
Source: Nat Commun. 2023 Jun 15;14:3556. doi: 10.1038/s41467-023-39201-1 (PMC10272158; doi:10.1038/s41467-023-39201-1)
Supplement: Supplementary file 3 — Description of Additional Supplementary Files [file 41467_2023_39201_MOESM3_ESM.pdf]

File name: Supplementary Movie 1

Description: Time evolution of the CALB : MHET complex along the unconstraint MD simulation at pH 5

File name: Supplementary Movie 2

Description: Time evolution of the CALB : MHET complex along the unconstraint MD simulation at pH 9
